# Biogeographic multi‐species occupancy models for large‐scale survey data

**DOI:** 10.1002/ece3.9328

**Published:** 2022-10-01

**Authors:** Jacob B. Socolar, Simon C. Mills, Torbjørn Haugaasen, James J. Gilroy, David P. Edwards

**Affiliations:** ^1^ Faculty of the Environment and Natural Resources Management Norwegian University of Life Sciences Ås Norway; ^2^ Ecology and Evolutionary Biology School of Biosciences, University of Sheffield Sheffield UK; ^3^ School of Environmental Sciences University of East Anglia Norwich UK; ^4^ Cornell Lab of Ornithology Cornell University Ithaca New York USA

**Keywords:** community model, hierarchical model, occupancy model, pooling, spatial scale

## Abstract

Ecologists often seek to infer patterns of species occurrence or community structure from survey data. Hierarchical models, including multi‐species occupancy models (MSOMs), can improve inference by pooling information across multiple species via random effects. Originally developed for local‐scale survey data, MSOMs are increasingly applied to larger spatial scales that transcend major abiotic gradients and dispersal barriers. At biogeographic scales, the benefits of partial pooling in MSOMs trade off against the difficulty of incorporating sufficiently complex spatial effects to account for biogeographic variation in occupancy across multiple species simultaneously. We show how this challenge can be overcome by incorporating preexisting range information into MSOMs, yielding a “biogeographic multi‐species occupancy model” (bMSOM). We illustrate the bMSOM using two published datasets: Parulid warblers in the United States Breeding Bird Survey and entire avian communities in forests and pastures of Colombia's West Andes. Compared with traditional MSOMs, the bMSOM provides dramatically better predictive performance at lower computational cost. The bMSOM avoids severe spatial biases in predictions of the traditional MSOM and provides principled species‐specific inference even for never‐observed species. Incorporating preexisting range data enables principled partial pooling of information across species in large‐scale MSOMs. Our biogeographic framework for multi‐species modeling should be broadly applicable in hierarchical models that predict species occurrences, whether or not false absences are modeled in an occupancy framework.

## Graphical abstract

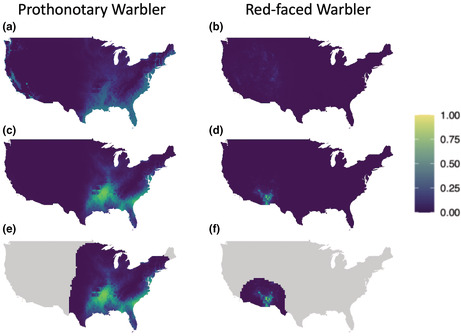

## 1 INTRODUCTION

Community ecologists often seek inference about occurrence patterns of multiple species simultaneously. To improve inference, especially for infrequently detected species, hierarchical models such as multi‐species occupancy models (MSOMs) share information across species via hierarchical random effects (Devarajan et al., 2020). MSOMs were originally developed for application to relatively homogeneous study areas where occupancy probabilities vary little across space (Dorazio & Royle, 2005). Subsequently, MSOMs have been applied to a variety of landscapes where occupancy probabilities are modeled as a function of site‐specific covariates (e.g., Ribeiro Jr. et al., 2018; Rich et al., 2017; Tingley & Beissinger, 2013).

At very large spatial scales that subsume biogeographic variation in species ranges, the biologically relevant covariate structure becomes exceedingly complex. The biogeographic distributions of multiple species depend on species‐specific interactions between numerous environmental, geographic, and historical factors. Rather than attempting to parameterize and fit these myriad effects, large‐scale single‐species distribution models often eschew the generalized linear modeling framework in favor of highly flexible additive effects (Rushing et al., 2020) or machine‐learning methods such as Maxent (Phillips et al., 2006) or regression trees (Fink et al., 2010). Such approaches, however, are not easily amenable to pooling information across data‐poor species for robust community‐level inference. As a result, there is a need for hierarchical multi‐species approaches to study community variation across biogeographic spatial scales (Janousek & Dreitz, 2020; Jarzyna & Jetz, 2018).

When the data at hand are insufficient to estimate realistic parametric models that adequately capture the biogeography of every species in a study region, the benefit of partial pooling across species trades off against the detriment of species‐specific spatial biases. Across complex biogeographic landscapes, we expect that models that fail to robustly account for species' ranges will tend to underestimate occupancy at points within a species' range and overestimate occupancy at points outside a species' range.

Given the difficulty of estimating complex biogeographic patterns in large‐scale MSOMs, previous authors have applied a variety of post hoc strategies to address inferential problems that arise from fitting simple MSOMs to biogeographically complex regions. For example, Jarzyna and Jetz (2018) applied a MSOM to predict terrestrial bird richness across the coterminous United States and manually adjusted their model output by setting occupancy probabilities to zero in regions where a species does not occur. Janousek and Dreitz (2020) applied a MSOM to the spatial complex bird communities of the greater Rocky Mountains of the United States, but 30 species (29%) failed a posterior predictive check and were excluded from further analysis. However, such statistical palliatives are not sufficient to ensure reliable inference, because post hoc exclusion of species or geographic ranges still allows poorly modeled data to inform inference in the remainder of the model.

Never‐observed species pose additional modeling challenges at biogeographic scales. In principle, traditional MSOMs can handle never‐observed species via data augmentation with excess pseudospecies, each of which is given an all‐zero detection history and is included or excluded from the true community according to a Bernoulli random variable with modeled probability Ω(Dorazio & Royle, 2005). However, these models cannot estimate independent covariate relationships for the never‐detected species, and some authors have chosen to exclude data‐augmented pseudospecies from downstream analyses (e.g., Tingley & Beissinger, 2013). Incorporating data‐augmentation approaches into models that leverage traits or phylogeny to predict detection (Sólymos et al., 2017) or occupancy (e.g., via trait–environment interactions) is especially challenging, requiring potentially dubious assumptions about the trait distributions for never‐detected species or the discretization of traits into functional guilds (Tenan et al., 2017).

Recent progress toward multi‐species pooling in biogeographic‐scale MSOMs, including models for never‐observed species, has focused on discretizing the study region into spatial units (Sutherland et al., 2016; Tobler et al., 2015) and discretizing the community into ecological guilds (Tenan et al., 2017). Sutherland et al. (2016) propose a multi‐region model where data‐augmented MSOMs are fit to each region and region‐specific community richness is directly modeled as a function of covariates. Importantly, the identities of species, including species that are never detected in a particular region, are fixed across regions, thus enabling pooled estimation of species‐specific occupancy and detection probabilities across the entire multi‐region study area. Tobler et al. (2015) fit a similar model without data augmentation, such that the potential pool of species in any region is exactly the total pool of species observed study‐wide, and all species identities are fixed and known. Tenan et al. (2017) extend the approach of Sutherland et al. (2016) to trait‐based models, discretizing the community into ecological guilds and estimating the richness of never‐observed species for each guild separately. However, all of these methods rely on the assumption that, conditional on covariates, the spatially discrete regions are internally homogeneous and mutually independent. Therefore, they are not suitable for application to biogeographic landscapes with complex and continuous spatial variation.

For many taxa, a wealth of preexisting biogeographic information is available in the form of range maps, geospatial sightings databases, and/or published range descriptions. We hypothesized that by leveraging this information, we could develop simple, tractable multi‐species models that yield reliable pooled inference about in‐range occupancy probabilities while avoiding the pitfall of conflating in‐range and out‐of‐range occupancy probabilities within and across species. We achieve such inference by collapsing complex, multidimensional biogeographic variation into simple summary covariates, which we call *range covariates*. Possible range covariates include (transformations of) the distance to the nearest geographic range margin or elevational range limit. Such information is increasingly available, especially for taxa amenable to occupancy modeling (Jetz et al., 2012). We refer to an MSOM that incorporates range covariates as a *biogeographic multi‐species occupancy model* (bMSOM). Like the multi‐region model of Tobler et al. (2015), the bMSOM fixes the identity of every species (including never‐observed species) in the dataset. However, unlike all previous models, the bMSOM handles arbitrarily complex biogeographic‐scale spatial dependencies using a very simple covariate structure.

Here, we formally describe the bMSOM and apply it to two published datasets: 51 Parulid warbler species in the United States Breeding Bird Survey (49 observed; two never‐observed), and 910 bird species in forests and pastures of Colombia's West Andes (314 observed, 596 never‐observed). In addition to providing a mechanism for principled data pooling across very large spatial scales, bMSOMs fix the identity of every species in the metacommunity and link those identities to real‐world species with known traits. They are therefore exceptionally suited to trait‐based models for occupancy and detection, analyses of point‐scale richness, and biogeographically pooled analyses of the influence of local‐scale environmental variation on community composition or structure. Furthermore, bMSOMs sometimes allow for a priori exclusion of data at extralimital sites (where occupancy is implausible), thereby reducing the total dataset size and the computational resources required for model fitting.

## 2 METHODS

### 2.1 Model formulation

We formulate the likelihood for the standard MSOM as

$$Y_{\mathrm{ijk}}∼\text{Bernoulli}\left({Z_{\mathrm{ij}}}^{*}\theta_{\mathrm{ijk}}\right)$$

$$Z_{\mathrm{ij}}∼\text{Bernoulli}\left(\psi_{\mathrm{ij}}\right)$$

$$\text{logit}\left(\psi \right)=a+\mathrm{Xb}$$

$$\text{logit}\left(\theta_{k}\right)=c+W_{k}d$$

$$\left[a,b,c,d\right]∼R$$

where *i*, *j*, and *k* index the species, site, and visit; ***Y*** is an array of binary detection/non‐detection data; ***Z*** is a matrix giving the latent true occupancy state; θ is an array of detection probabilities conditional on occupancy (i.e., $p\left(Y_{\mathrm{ijk}}=1|Z_{\mathrm{ij}}=1\right)$), such that $\theta_{k}$ is a matrix of detection probabilities for the kth visit; ψ is a matrix of occupancy probabilities (i.e., $p\left(Z_{\mathrm{ij}}=1\right)$); ***a*** and ***c*** are column vectors of intercepts for occupancy and detection, respectively; ***X*** and ***W*** are design matrices for occupancy and detection, respectively; ***b*** and ***d*** are column vectors of coefficients for occupancy and detection, respectively; and *R* is the joint random effects distribution for *a*, *b, c*, and *d*. At a minimum, *R* must include random intercepts by species for both occupancy (***a***) and detection (***c***).

### 2.2 The bMSOM

The likelihood for the bMSOM is no different from the standard MSOM; what differs is the data. As in a data‐augmented MSOM (Dorazio & Royle, 2005), we append all‐zero detection histories for never‐observed species. However, each of these all‐zero entries corresponds to a specific species that we know a priori occurs in the biogeographic vicinity of the sampling points, and so we treat all species as present in the metacommunity and “available” for occupancy.

Crucially, we include one or more “range covariates” that describe whether a given species is in‐range or out‐of‐range at each point, and we estimate species‐specific random coefficients for the range covariates. For example, if species‐specific minimum and maximum elevation data are available along an elevational gradient, an appropriate range covariate might be the squared elevational distance from a survey point to the midpoint of a species' elevational range. If species differ substantially in their elevational breadth, we might rescale these differences for each species separately prior to squaring, such that values of 1 correspond to the species‐specific upper range limits and values of −1 correspond to the species‐specific lower range limits. When species are distributed over two‐dimensional space rather than along one‐dimensional gradients, we suggest using a range covariate based on the geographic distance from a survey point to the species geographic range margin. When only crude range descriptions are available, the range covariate might simply be binary, designed to distinguish areas that are clearly out‐of‐range. Regardless of the precise nature of the range covariates, we include them in the bMSOM with species‐specific random slopes.

In the bMSOM context, it is sometimes additionally useful to completely exclude severely out‐of‐range species‐site combinations from analysis, a process that we call “*biogeographic clipping*.” By excluding sites where occupancy probabilities are a priori negligible, it is possible to improve within‐range estimation while reducing the computational burden of model fitting. For example, biogeographic clipping can account for sharp range margins associated with abrupt biogeographic barriers (e.g., mountains or deepwater marine barriers) while still allowing occupancy probabilities to decay more gradually at range margins elsewhere. Likewise, migratory species can be modeled with temporal clipping, where species‐site combinations are removed from the data if the site was surveyed outside of the dates of potential presence. Here we assume that repeat visits to a site occur sufficiently quickly to avoid problems of closure for migratory species.

### 2.3 Example 1: Warblers of the coterminous United States

Jarzyna and Jetz (2018) analyzed multi‐species occupancy patterns of North American birds by applying a traditional MSOM to the United States Breeding Bird Survey (BBS) dataset (Bystrak, 1981). With a focus on the year 2018, the coterminous United States, and the Parulid warblers, we reimplemented the modeling framework of Jarzyna and Jetz (2018) and compared the traditional MSOM with the bMSOM. We restricted the analysis to Parulid warblers (as opposed to the full North American avifauna) for the sake of computational efficiency, and we selected the Parulid warblers in particular because they are relatively speciose (51 species breed in the coterminous United States), display marked variation in species' ranges, are well sampled by BBS protocols, and are sufficiently homogeneous in their territoriality and vocal behavior to ensure that they approximately satisfy exchangeability assumptions for hierarchical modeling, even in the absence of species‐specific covariates.

We downloaded BBS data for the year 2018 from www.pwrc.usgs.gov, and we obtained range maps for all Parulid warblers that regularly breed within 200 km of the coterminous United States from Birdlife International (BirdLife International and Handbook to the Birds of the World, 2019). To develop a range covariate, we measured the distance from the starting point of each BBS survey route to the nearest edge of each species' range, excluding range limits associated with shorelines. We then sought a transformation of the species ranges that would approximately linearize the logit‐proportion of occupied points. We believed a priori that the function should asymptote at large negative distances (i.e., in the core of the range), and we sought a function that would asymptote at zero, such that hierarchical model components would effectively be setting a prior on occupancy probabilities in the core of a species range. We believe that an asymptote at zero should help to ensure exchangeability across species and should aid in eliciting informative priors (if desired).

We binned all BBS point‐species combinations by their distance to the range edge (negative distances at in‐range points, positive distance at out‐of‐range points), and we examined several functions to select one that approximately linearizes the logit‐proportion of occupied points, ultimately selecting the inverse logit of distance‐to‐range expressed in units of 200 km (Supporting Information, section 1).

We fit three occupancy models to these data. Model 1 (traditional MSOM) is the model of Jarzyna and Jetz (2018), with correlated random intercepts for detection and occupancy and a random slope for the effect of elevation on occupancy (Zipkin et al., 2009):

$$\begin{array}{c} \log\mathrm{it}\left(\psi_{\mathrm{ij}}\right)=a_{i}+\beta_{1i}{\text{elev}}_{j}\\ \log\mathrm{it}\left(\theta_{i}\right)=c_{i} \end{array}$$

Model 2 (bMSOM) extends Model 1 via the inclusion of the range covariate described above, to become:

$$\log\mathrm{it}\left(\psi_{\mathrm{ij}}\right)=a_{i}+\beta_{1i}{\text{elev}}_{j}+\beta_{2i}{\text{distanceToRange}}_{\mathrm{ij}}$$

Model 3 (bMSOM with biogeographic clipping) is equivalent to model 2 but excluding all species‐point combinations >400 km from the mapped range, a distance beyond which no detections existed in the data.

We compared the predictive performance of models 1, 2, and 3 via approximate leave‐one‐out cross‐validation using Pareto‐smoothed importance sampling with moment‐matching (Vehtari et al., 2017, 2021). For each pair of models, we compared overall predictive performance as well as predictive performance for each species separately. For models 1 and 2, we compared predictive performance over all points as well as over just the subset of points that we retained after biogeographic clipping. For comparisons involving model 3, we evaluated predictions only over the subset of points retained after biogeographic clipping, as this model sees nondetections outside of the clipped range as deterministic structural zeros.

Code to perform these analyses is available online at https://github.com/jsocolar/bmsom_paper/tree/master/BBS.

### 2.4 Example 2: Forest conversion in Colombia's West Andes

We applied the bMSOM to a dataset of bird species at 146 point‐count stations on an elevational gradient from 1260 to 2680 masl in Colombia's West Andes. Each point was visited on four consecutive or nearly‐consecutive days (Gilroy, Edwards, et al., 2014; Gilroy, Woodcock, et al., 2014). Points were located in either forest or pasture and were arranged in clusters of three points each nested inside one of three subregions. Following the taxonomy of BirdLife International, 910 bird species potentially occurred in the vicinity of the points, based on biogeographic clipping (see below). Of these, 314 were detected at least once and 596 were never observed. Our inferential goal was to assess how point‐scale species richness varies along the elevational gradient.

We implemented a biogeographically clipped bMSOM framework to address Colombia's exceedingly complex biogeography. We incorporated two range covariates, based on elevation and geography. The elevational range covariate was based on the elevational limits reported in Ayerbe Quiñones (2018), supplemented with several additional references for species whose taxonomic treatment differed between Ayerbe Quiñones (2018) and BirdLife International (2020). We standardized the elevations of each point across species by linearly rescaling the raw elevations of the points such that an elevation of 1 corresponded to the upper range limit, and an elevation of −1 corresponded to the lower range limit (Figure S1). We implemented biogeographic clipping at species‐standardized elevations of −3 and 3, beyond which our dataset contained no detections. We additionally implemented temporal clipping for migratory species, treating all migrants as deterministically absent outside of their normal dates of presence in Colombia (Supplementary Data).

We derived the geographic range covariate using digital range maps from Ayerbe Quiñones (2018; see also Vélez et al., 2021). We implemented biogeographic clipping at a buffer of 160 km around these maps, as well as at the crest of the West Andes and the floor of the Cauca Valley to address the complex biogeography of birds in this region. Against this biogeographic clipping, our field data exposed errors of omission in just two species, indicative of the high quality of the Ayerbe Quiñones maps. For these two species, we added range around previously known clusters of records (eBird, 2021) that coincided with the records in our data, and we refined the biogeographic clipping to incorporate an appropriate buffer around this additional range (see Supporting Information, section 2). The spatially balanced sampling of forest and pasture ensures that this post hoc addition of range does not compromise inference about responses to deforestation. Our field data exposed no errors of omission against our temporal clipping.

After performing all clipping, we selected an appropriate transformation of raw distance for the geographic range covariate following the procedure described for warblers above. Again, we approximately linearized the logit‐proportions by taking the inverse logits of the distance, this time measured in units of approximately 14.9 km (Supporting Information, section 1).

We modeled occupancy on the logit scale based on an intercept and coefficients for the geographic range covariate, the elevational range covariate (linear and quadratic terms), interactions between the elevational range covariate (linear and quadratic terms) and whether the species occurs at lowland elevations, land‐use, 18 species traits (Table S1), and the interactions of those 18 traits with land use. Our random effects structure incorporated random taxonomic intercepts for species and family, random spatial intercepts for species:cluster and species:subregion, and random taxonomic coefficients for all range covariates (species‐specific terms) and for land use (species‐ and family‐specific terms). Occupancy is therefore:

$$\begin{array}{c} \log\mathrm{it}\left(\psi_{\mathrm{ij}}\right)=a_{\mathrm{ij}}+\beta_{1i}\mathrm{ele}v_{j}+\beta_{2i}\mathrm{ele}v_{j}^{2}+\beta_{3i}\text{habita}t_{j}+\beta_{4i}\text{distanceToRang}e_{\mathrm{ij}}+\beta_{5}\text{lowlan}d_{i}\\ +\beta_{6}\left(\mathrm{ele}v_{j}\times \text{lowlan}d_{i}\right)+\beta_{7}\left(\mathrm{ele}v_{j}^{2}\times \text{lowlan}d_{i}\right)+\beta_{8}\text{mtnBarrie}r_{i}+\beta_{9}\text{valBarrie}r_{i}\\ +\beta_{10}\text{elevMedia}n_{i}+\beta_{11}\text{elevBreadt}h_{i}+\beta_{12}\text{forestPresen}t_{i}+\beta_{13}\text{forestSpecialis}t_{i}\\ +\beta_{14}\text{tfSpecialis}t_{i}+\beta_{15}\text{dryForestPresen}t_{i}+\beta_{16}\text{floodDrySpecialis}t_{i}\\ +\beta_{17}\text{aridPresen}t_{i}+\beta_{18}\text{migrator}y_{i}+\beta_{19}\left(\text{elevMedia}n_{i}\times \text{forestPresen}t_{i}\right)\\ +\beta_{20}\left(\text{elevMedia}n_{i}\times \text{forestSpecialis}t_{i}\right)+\beta_{21}\mathrm{mas}s_{i}+\beta_{22}\text{dietInver}t_{i}\\ +\beta_{23}\text{dietCar}n_{i}+\beta_{24}\text{dietFruitNec}t_{i}+\beta_{25}\text{dietGra}n_{i}+\beta_{26}\left(\text{habita}t_{j}\times \text{mtnBarrie}r_{i}\right)\\ +\beta_{27}\left(\text{habita}t_{j}\times \text{valBarrie}r_{i}\right)+\beta_{28}\left(\text{habita}t_{j}\times \text{elevMedia}n_{i}\right)\\ +\beta_{29}\left(\text{habita}t_{i}\times \text{elevBreadt}h_{i}\right)+\beta_{30}\left(\text{habita}t_{j}\times \text{forestPresen}t_{i}\right)\\ +\beta_{31}\left(\text{habita}t_{j}\times \text{forestSpecialis}t_{i}\right)+\beta_{32}\left(\text{habita}t_{j}\times \text{tfSpecialis}t_{i}\right)\\ +\beta_{33}\left(\text{habita}t_{j}\times \text{dryForestPresen}t_{i}\right)+\beta_{34}\left(\text{habita}t_{j}\times \text{floodDrySpecialis}t_{i}\right)\\ +\beta_{35}\left(\text{habita}t_{j}\times \text{aridPresen}t_{i}\right)+\beta_{36}\left(\text{habita}t_{j}\times \text{migrator}y_{i}\right)\\ +\beta_{37}\left(\text{habita}t_{j}\times \mathrm{mas}s_{i}\right)+\beta_{38}\left(\text{habita}t_{j}\times \text{dietInver}t_{i}\right)+\beta_{39}\left(\text{habita}t_{j}\times \text{dietCar}n_{i}\right)\\ +\beta_{40}\left(\text{habita}t_{j}\times \text{dietFruitNec}t_{i}\right)+\beta_{41}\left(\text{habita}t_{j}\times \text{dietGra}n_{i}\right) \end{array}$$

We modeled detection on the logit scale based on an intercept and coefficients for land use, time of day (given as hours post‐sunrise), four species traits, and the interaction between time of day and the median elevation where a species occurs. Our random effects structure incorporated random taxonomic intercepts for species and family, a random intercept for species:observer, and random taxonomic coefficients for time of day (species‐specific terms) and land use (species‐ and family‐specific terms):

$$\begin{array}{c} \log\mathrm{it}\left(\theta_{\mathrm{ijk}}\right)=c_{\mathrm{ijk}}+\delta_{1i}\text{habita}t_{j}+\delta_{2i}\mathrm{tim}e_{\mathrm{jk}}+\delta_{3}\mathrm{mas}s_{i}+\delta_{4}\text{elevMedia}n_{i}+\\ \;\delta_{5}\text{migrator}y_{i}+\delta_{6}\text{dietCar}n_{i}+\delta_{7}\left(\mathrm{tim}e_{\mathrm{jk}}\times \text{elevMedia}n_{i}\right) \end{array}$$

See Supporting Information (section 3) for details of our prior specification.

To assess our ability to recover principled trait‐based estimates of sensitivity to deforestation, including for never‐observed species, we compared the sensitivity estimates from the model (i.e., the coefficients for the forest/pasture term) against independently estimated forest dependency scores from BirdLife International (2020). We repeated this comparison for just species with at least one observation in our data and for just the never‐observed species.

We then use the bMSOM to predict the local species richness (including never‐observed species) in forest and pasture across an elevational gradient in the Colombian West Andes. For comparison, we also estimated species richness along the elevational gradient in forest and pasture using a data‐augmented multi‐species occupancy model including the 314 observed species and 1000 never‐observed pseudospecies (Dorazio & Royle, 2005). To enable use of the data‐augmented model, we removed all species‐specific covariates (including information about range, traits, dates of occurrence, and family‐level classification) from the analysis.

### 2.5 Model fitting

We implemented occupancy models using Hamiltonian Monte Carlo sampling in Stan (Stan Development Team, 2021) via R packages brms (Bürkner, 2017) for the Parulid warblers and cmdstanr (Gabry & Češnovar, 2021) for the Colombian birds. We performed model comparison using the R package “loo” (Vehtari et al., 2020). For all warbler models, we ran four chains for 1000 warmup iterations and 1000 sampling iterations. For the Colombian Andes, we ran four chains for 1500 warmup iterations and 1500 sampling iterations. For the data‐augmented model, we encountered substantial challenges in model fitting; we describe these problems and their resolution in the Supporting Information (section 4). We ensured that all models (except the data‐augmented model; see Supporting Information, section 4) converged with maximum r‐hats less than 1.03 for all parameters, no divergences in the Hamiltonian trajectories, and energy fraction of missing information greater than 0.2 in all chains.

Code to perform these analyses is available online at https://github.com/jsocolar/bmsom_paper/tree/master/wAndes.

## 3 RESULTS

### 3.1 Warblers of the coterminous United States

**FIGURE 1:**
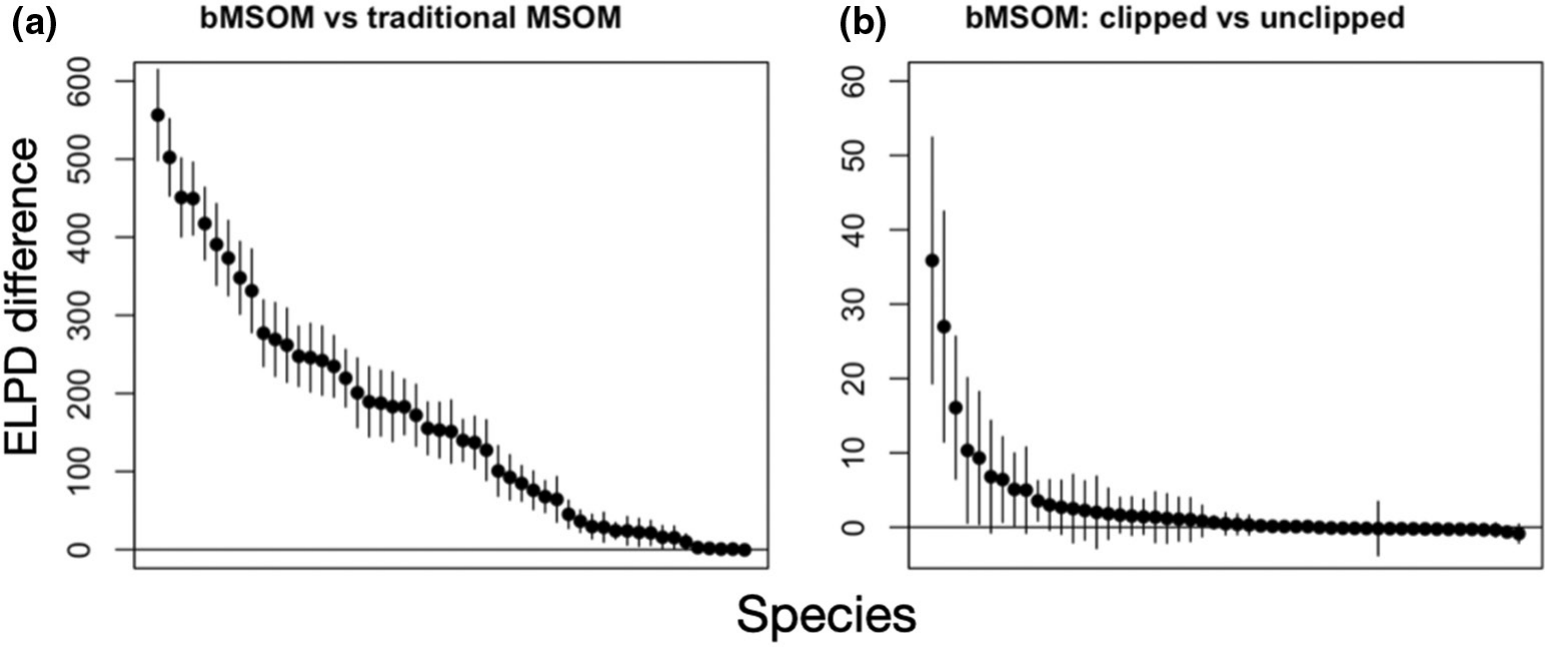
Differences in species‐specific expected log pointwise density (ELPD) calculated by approximate leave‐one‐out cross validation for the BBS data. Points represent species‐specific posterior means and are ordered by decreasing ELPD difference; lines represent ±2 standard errors. Positive values indicate superior predictive performance in the first model. Comparisons are performed across all points (a) or across just the subset of points that are retained in the clipped model (b).

The inclusion of the distance covariate in the bMSOM yielded dramatic improvements in predictive performance (Figure 1), with an improvement in expected log predictive density (ELPD) of 8558 with standard error (SE) 127. Predictive performance improved for 50 out of 51 species, the only exception being a marginal decrease of −0.4 (SE 0.8) for Tropical Parula (Figure 1a).

Biogeographic clipping delivered further gains in predictive performance at in‐range points, with an ELPD improvement of 147 (SE 17). Within the species‐specific regions that were retained after clipping, predictive performance improved for 33 out of 51 species (Figure 1b). Among the 18 species that did not see improvements, we observed the largest decrease in ELPD in Lucy's Warbler, but even this decrease was only marginal (−0.9; SE 0.6), which is too small to conclude that the clipped model performs worse than the unclipped model for Lucy's Warbler or any other species. Biogeographic clipping also yielded substantial gains in computational efficiency, reducing the runtime by a factor of almost three, from a mean per‐chain execution time of 5.8 h (worst‐case chain 6.1 h) to 2.0 h (worst‐case chain 2.1 h) on an M1 Macbook Air.

**FIGURE 2:**
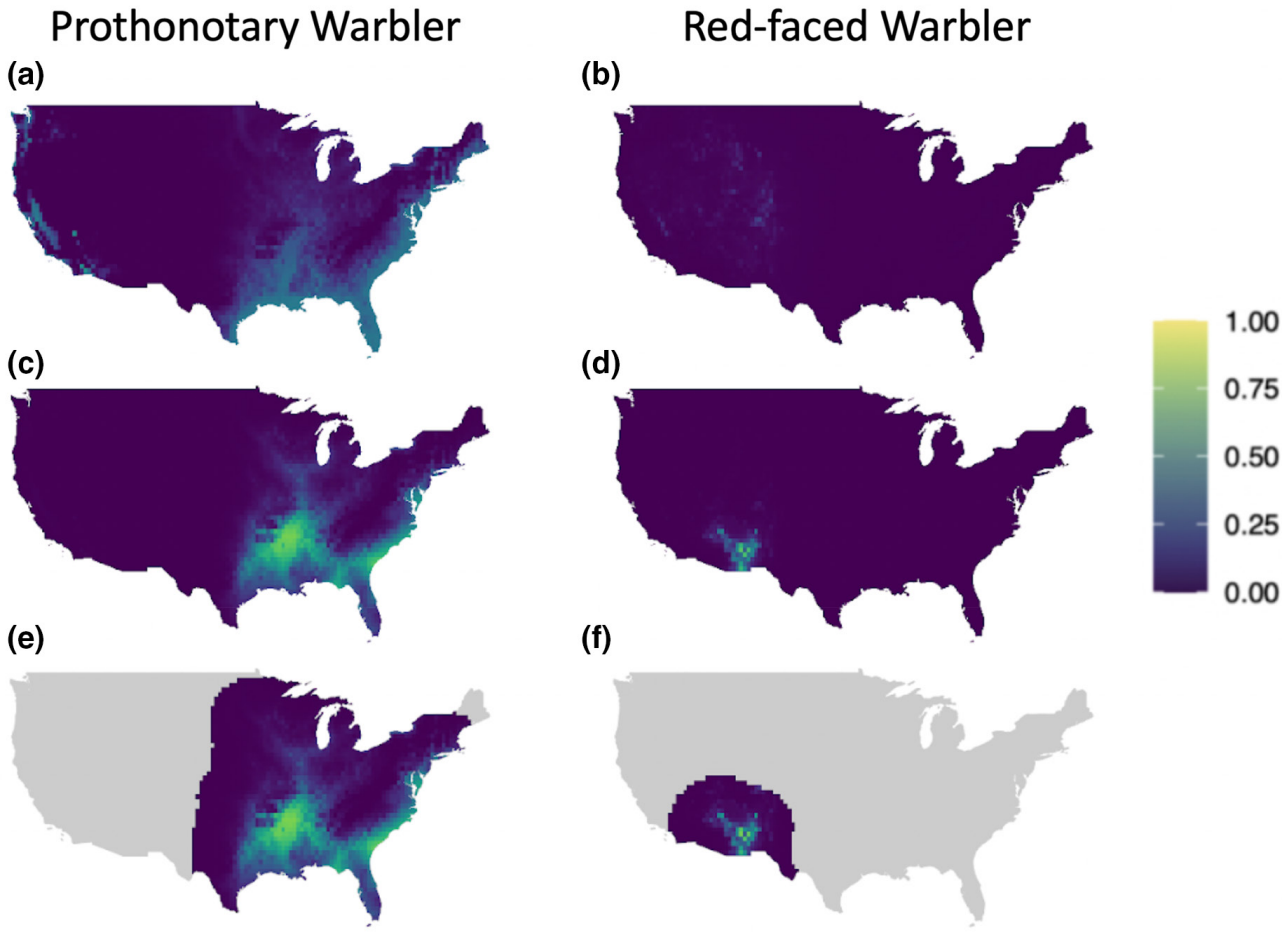
Predicted occupancy probabilities for prothonotary warbler (left‐hand column), a low‐elevation eastern species, and red‐faced warbler (right‐hand column), a high‐elevation southwestern species. Predictions are given by the traditional MSOM (a, b), the bMSOM (c, d), and the clipped bMSOM (e, f). Equivalent figures for all species are available in Figure S2.

**FIGURE 3:**
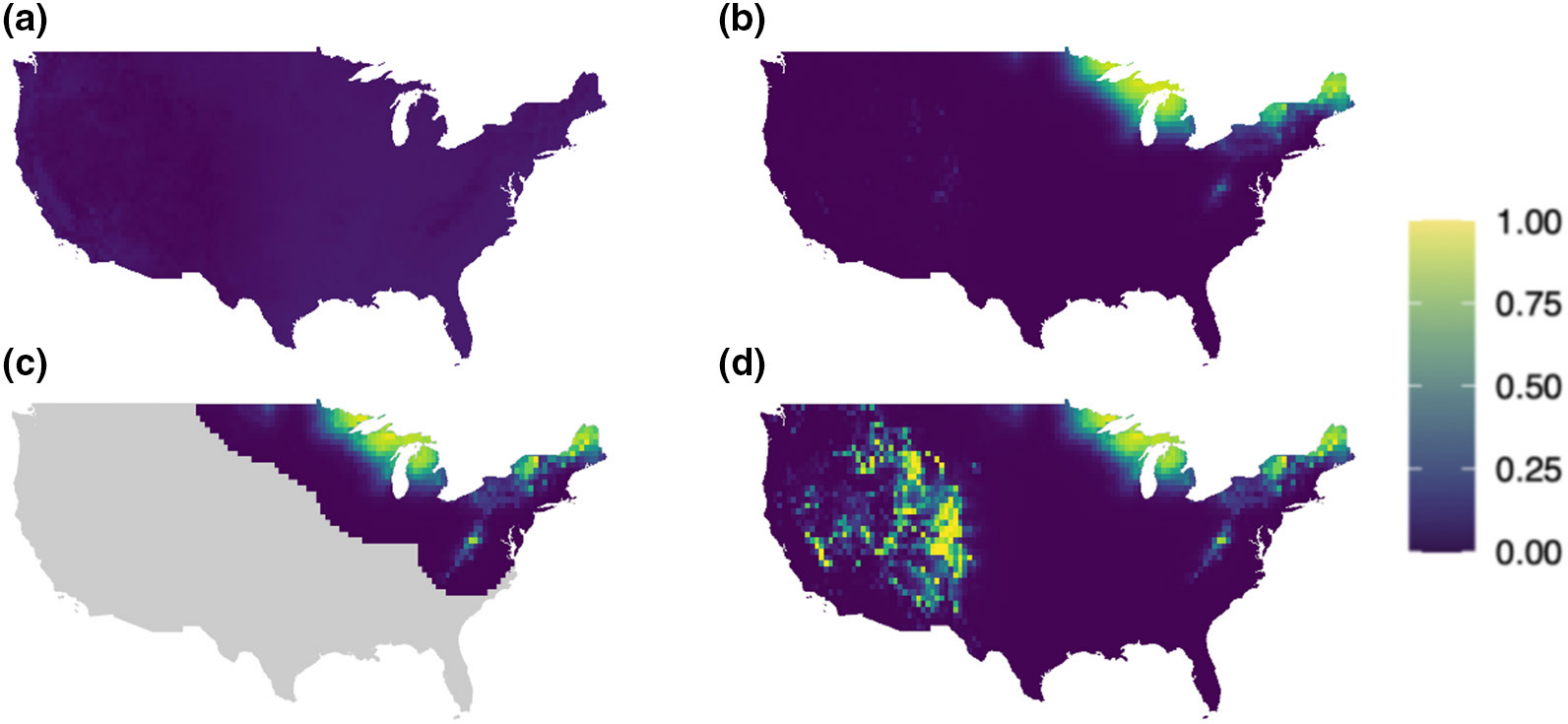
Predicted occupancy probabilities for mourning warbler from (a) the traditional MSOM, (b) the bMSOM without clipping, (c) the clipped bMSOM, and (d) the clipped bMSOM coefficients projected across the entire country. Mourning warbler tends to occur at higher elevation within its eastern range. Biogeographic clipping allows the model to estimate appropriately strong elevational relationships in the eastern United States, without the need to avoid predicting high occupancy probabilities across the mountainous western United States. In (d) the steep elevation relationship overcomes the negative relationship with geographic distance to yield high occupancy probabilities in the mountains of the western United States.

Predicting the traditional MSOM across space yielded relatively uniform occupancy probabilities compared with the bMSOM, which universally predicted higher occupancy probabilities at in‐range points and lower probabilities at out‐of‐range points (Figure 2). The predictions of the clipped bMSOM were generally quite similar to those of the unclipped bMSOM, though small differences were apparent for some species. In these cases, the clipped model tended to estimate steeper elevational relationships, which reflects the clipped model's flexibility to fit locally appropriate relationships unconstrained by the need for accurate prediction at severely out‐of‐range sites. For example, the Mourning Warbler is restricted to the eastern United States, and within this range, it tends to occur at high elevations. Without clipping, the bMSOM estimates only a modest positive elevational relationship (1.4, 95% CI 0.8–2.0), because steeper estimates yield unacceptably high occupancy probabilities in the high‐elevation mountains of the western United States (Figure 3). Clipping allows the model to estimate an appropriately steep relationship (3.2, 95% CI 2.1–4.4) within the species' northeastern range. We provide maps of predicted occupancy probabilities for all species in Figure S2.

### 3.2 Forest conversion in Colombia's West Andes

**FIGURE 4:**
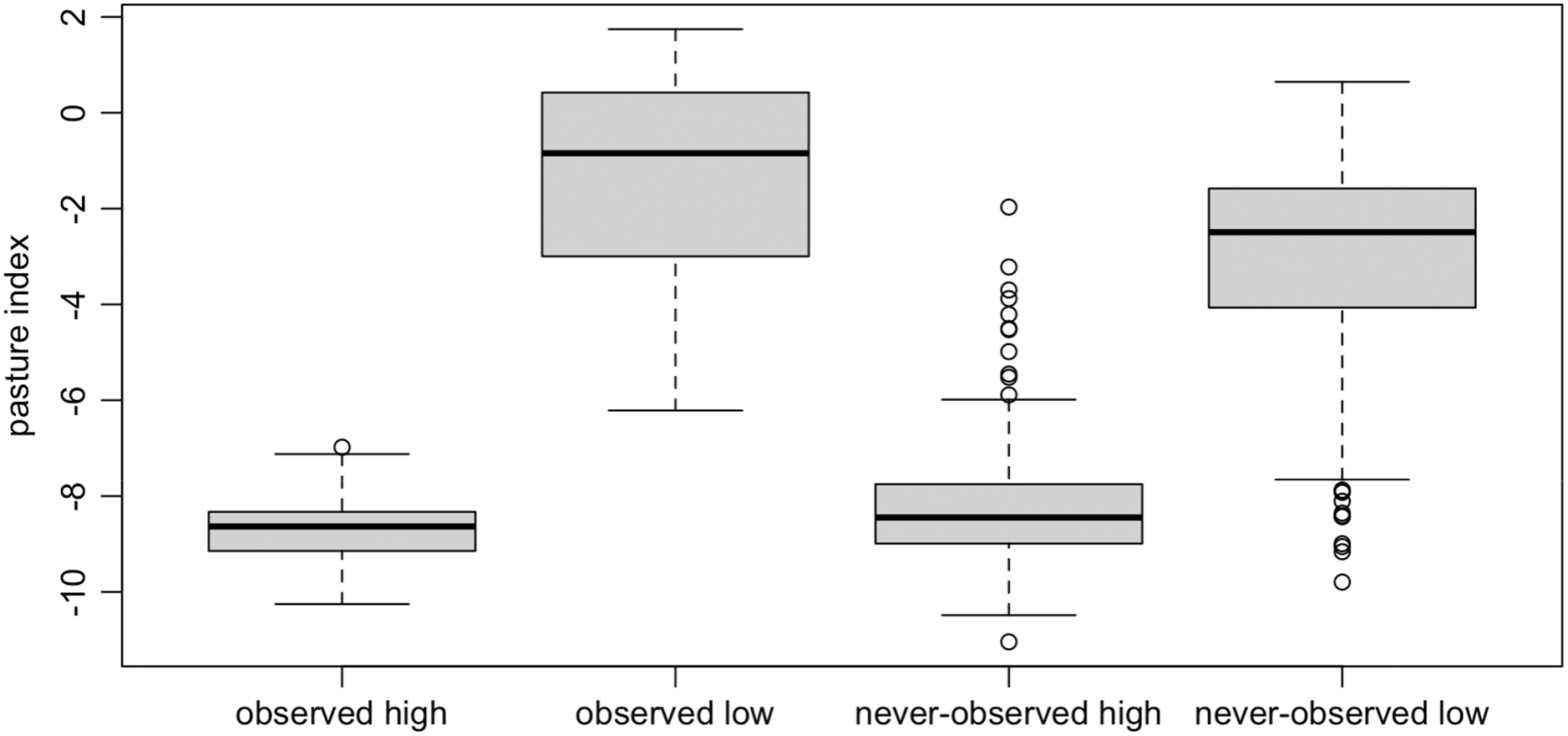
Model‐estimated logit‐scale effects of forest conversion to pasture on avian occupancy in the West Andes. The pasture index is the difference in the log‐odds of occupancy between forest and pasture. Data points are posterior medians for individual species. Box plots are grouped by whether or not the species was detected at least once during sampling (observed/never‐observed), and by whether BirdLife international independently ascribes the species to the “high” forest dependency category (high) or to the “low” or “not a forest species” categories (low). Species ascribed to “medium” or “unknown” forest dependency categories are omitted from this figure.

The bMSOM yielded reliable trait‐based inference for the sensitivity of the entire avifauna, including never‐observed species (Figure 4). We provide a summary of the fitted model posterior in Table S1. Among species with at least one observation and classified by BirdLife International as having either high forest dependence or low/no forest dependence, the model universally inferred that species classified as highly forest‐dependent responded more negatively to forest conversion than other species. Even among species with no observations, the model successfully inferred that the vast majority of species classified as having high forest dependence respond more negatively to pasture than species with low/no forest dependence (Figure 4). Moreover, some of this overlap may arise due to the difficulty of accurately categorizing forest dependence in rarely encountered species.

**FIGURE 5:**
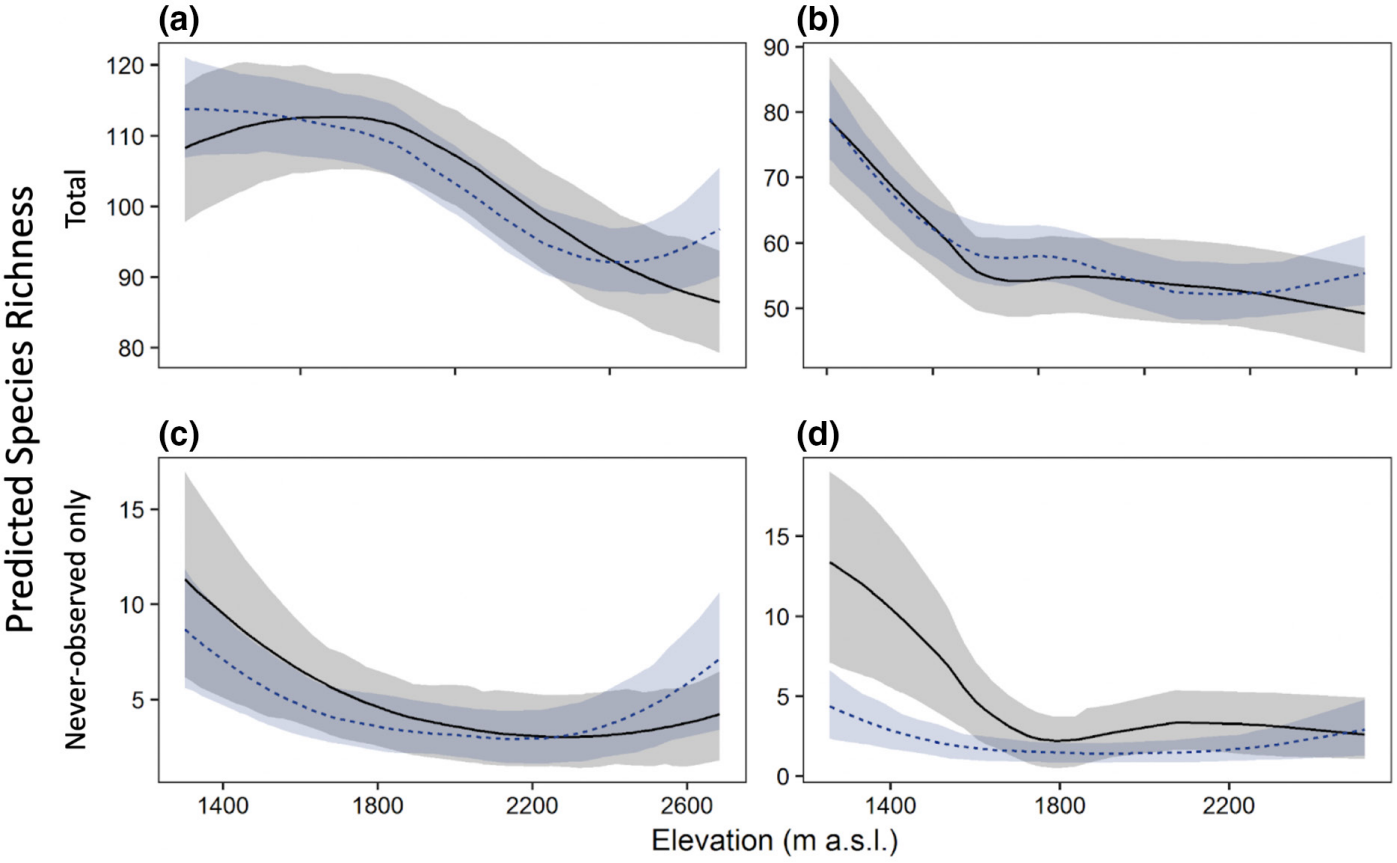
Predicted point‐scale avian richness for all species (a and b) and only never‐observed species (c and d) along the west Andean elevational gradient in forest (a, c) and pasture (b, d). The bMSOM is shown in gray and the data augmented MSOM is shown in blue with 80% credible interval overlaid. The data‐augmented model spuriously infers an excess of never‐observed species at points on the upper end of the elevational gradient.

The bMSOM provided species‐specific trait‐based inference on occupancy probabilities in both pasture and forest, even for never‐observed species. While the data‐augmented MSOM predicted similar patterns of alpha‐diversity along the gradient (Figure 5a,b), the data‐augmented model also displayed specific pathologies that affect both its practicality as an inferential tool and the quality of the resulting inference. First, the data‐augmented MSOM required dramatically more computational resources and fine‐tuning of algorithmic parameters to successfully fit (Supporting Information, section 4). Second, the data‐augmented model implausibly estimated that species were included in the metacommunity with probability near unity (95% credible interval 0.995–1.000). The data‐augmented model accounts for the non‐detection of the 1000 never‐observed pseudospecies species by ascribing to them extreme elevational ranges that overlap little with the sampling points. Thus, the data‐augmented model predicts that never‐observed species occur most frequently at both the lower and upper extremes of the gradient (Figure 5c,d). At the lower end of the gradient, this pattern is expected based on the tendency for species richness to increase with increasing productivity and forest stature toward lower elevations and is consistent with the predictions of the bMSOM. At the upper end, however, this pattern is at odds with both theoretical expectations and with the predictions of the bMSOM. In particular, the data‐augmented model estimates a spurious increase in alpha richness near the highest sampling points (all of which are in forest; Figure 5a) due to an uptick in occupancy of never‐observed species (Figure 5c).

## 4 DISCUSSION

Ecologists increasingly seek inference about occurrence patterns for multiple species over vast spatial scales (Janousek & Dreitz, 2020; Jarzyna & Jetz, 2018). We show that it is possible to leverage the existing multi‐species occupancy model framework to provide principled inference over these large scales simply by including covariates that summarize the positions of sampling points with respect to species ranges. When appropriate range information is available, the resulting biogeographic models deliver improved predictive performance, trait‐based inference for unobserved species, and computational speed‐up compared with traditional approaches. The application of traditional MSOMs (that lack range covariates) at biogeographic spatial scales results in lack‐of‐fit and unrealistic spatial predictions, suggesting that modelers must proceed with great caution when fitting large‐scale MSOMs in contexts where a priori range information is unavailable.

### 4.1 Core advantages of the bMSOM

There are three main advantages of bMSOM over traditional MSOMs. First, we get better inference on the observed species for a range of reasons. It is unsurprising that the introduction of a covariate that reliably distinguishes in‐range from out‐of‐range points should improve predictive performance, but the full scope of the improvement is broad and at times subtle. For example, although revising extralimital occupancy probabilities to zero after model fitting eliminates obviously erroneous predictions of extralimital occupancy, doing so does not enable accurate estimation of within‐range occupancy probabilities (Jarzyna & Jetz, 2018). The traditional MSOM's conflation of occupancy probabilities at in‐range and out‐of‐range points induces a strong negative bias in occupancy probabilities at in‐range points (Figure 2). Range covariates additionally improve predictive performance by placing species on a common scale, where exchangeability assumptions are more likely to hold. For instance, by using species‐standardized elevations to model avian occupancy in Colombia's West Andes, we ensured that the magnitudes of the quadratic coefficients are likely to be similar across species, irrespective of heterogeneity in elevational range breadth. In traditional MSOMs, heterogeneity in elevational range breadth might be confounded with phylogeny, traits, or other predictors of interest, which could impede clear inference about covariate relationships (Sólymos et al., 2017).

Likewise, the geographic range covariates ensure that the intercepts for all species correspond to occupancy probabilities in their core ranges, partially removing potential relationships between intercepts and range size. In the BBS analysis, the bMSOM yielded large gains in predictive performance even for the most widespread species in the dataset, the Common Yellowthroat (ELPD gain 153, SE 18). Part of this effect results from principled handling of range margins in southern Texas and California, but part arises from better pooling of the intercept across species. Whereas the traditional occupancy model pools the intercept for Common Yellowthroat with intercepts for other species that reflect a mixture of in‐range and out‐of‐range occupancy probabilities, the bMSOM pools the intercept for Common Yellowthroat with intercepts that reflect in‐range occupancy probabilities across all species. Thus, the bMSOM estimates a larger intercept for Common Yellowthroat than the naïve MSOM, yielding better predictive performance.

A second key advantage of incorporating range covariates into occupancy models is their ability to specify both the metacommunity size and the identity of every species in the metacommunity. Doing so simplifies the formulation and implementation of models that include never‐detected species, enables the use of species‐specific covariates to model occupancy for never‐detected species, and eliminates uncertainty in the total metacommunity size as a source of uncertainty in point‐level species richness. Thus, the bMSOM ameliorates the substantial computational challenges associated with fitting the data‐augmented model, the limitations on inference about never‐detected species inherent to the data‐augmented model (Tingley & Beissinger, 2013), and a variety of pathologies that arise in the data‐augmented model when detection probabilities are low (see Tingley et al.,  2020). At the same time, the bMSOM avoids assuming that variation in occupancy across space or in trait distributions affecting occupancy or detection is readily discretized (Sutherland et al., 2016; Tenan et al., 2017). In our analysis of the West Andean avifauna, the bMSOM was able to recover the forest dependency of never‐observed bird species with high fidelity (Figure 4). By leveraging this ability, we were able to predict alpha‐richness in forest and pasture for the full community using a procedure that avoided predicting spurious patterns among never‐observed species (Figure 5) and was not subject to the computational challenges of fitting the data‐augmented model (Supporting Information, section 4).

A third benefit associated with range covariates is the ability to perform biogeographic clipping, which can substantially reduce the computational burden of model fitting (a threefold reduction in our BBS analysis) and can improve model fit at biologically relevant sites (Figure 3). Biogeographic clipping also enables modelers to account for biogeographic barriers that produce abrupt drops in occupancy probability, with zero occupancy probability on one side of the barrier. Such drops are difficult to capture with general‐purpose range covariates that must also account for the more gradual decay in occupancy probability at other range margins. By removing species‐point combinations on the wrong side of biogeographic barriers from analysis, these out‐of‐range detection histories do not propagate (mis)information about the distance‐decay in occupancy probabilities near mapped range margins elsewhere.

### 4.2 Application in practice and conclusions

The importance of prior range information highlights one potential pitfall in occupancy modeling (including the traditional occupancy model) at scale: when is a range map good enough? While a range map does not need to precisely reflect range margins, significant errors of omission will carry through to posterior inference with zero occupancy probabilities at locations where a species is in fact present. In our West Andes dataset, for example, we identified deficiencies for a minority of species (*n* = 2), requiring manual adjustment of range maps to bring them up to date with known species' occurrences. While expert knowledge can be harnessed both to assess the quality of range maps as well as make any requisite changes, this does raise the danger of inflated “researcher degrees of freedom” (Simmons et al., 2011), and manual updating of range maps based on the observed data. Judicious care is needed to ensure that these choices do not generate unfounded inference.

Overall, the bMSOM carries advantages that are especially well suited for estimating covariate relationships by pooling across species with disparate ranges, uncovering local‐scale covariate relationships while controlling for broad‐scale biogeography, estimating alpha‐scale species richness, and trait‐based modeling of never‐observed species. On the other hand, due to the requirement for preexisting range data the bMSOM is ill suited for exploratory species‐distribution modeling at biogeographic scales or inference about the effects of environmental predictors that are spatially autocorrelated over scales comparable to species entire ranges. We caution, however, that except in data‐rich contexts where ranges can be reliably estimated from data for all species under study (and thus multispecies approaches are unlikely to be necessary or useful), approaches that do not incorporate range information are likely to yield poor inferences about occupancy, biasing in‐range occupancy probabilities downward while also predicting non‐negligible occupancy in many areas far removed from a species' range. There thus appear to be general limitations to the application of MSOMs at large spatial scales that subsume significant biogeographic turnover. In the absence of major sampling efforts that allow range‐wide variation in occupancy to be estimated from the data directly, application of occupancy models to species or taxonomic groups for which range information cannot be included will typically yield poor inference.

## AUTHOR CONTRIBUTIONS

**Jacob Socolar:** Conceptualization (equal); data curation (equal); formal analysis (equal); investigation (equal); methodology (equal); software (equal); visualization (equal); writing – original draft (equal); writing – review and editing (equal). **simon mills:** Conceptualization (equal); data curation (equal); formal analysis (equal); investigation (equal); methodology (equal); software (equal); visualization (equal); writing – review and editing (equal). **Torbjørn Haugaasen:** Conceptualization (supporting); data curation (supporting); funding acquisition (equal); investigation (supporting); project administration (equal); resources (equal); supervision (equal); writing – review and editing (supporting). **James J. Gilroy:** Data curation (supporting); investigation (equal); resources (supporting); writing – review and editing (supporting). **David P Edwards:** Conceptualization (supporting); data curation (supporting); funding acquisition (equal); investigation (supporting); project administration (equal); resources (equal); supervision (equal); writing – review and editing (supporting).

## CONFLICT OF INTEREST

The authors declare no conflict of interest.

## Supporting information


**Appendix S1** Supporting Information

## Data Availability

Data for the west Andes analyses are available in Figshare at 10.6084/m9.figshare.20400687. Data from the USGS Breeding Bird Survey are publically available and are directly downloaded in the supplementary code. Code is available at https://github.com/jsocolar/bmsom_paper.
