# Supplementary material for: Biogeographic multi‐species occupancy models for large‐scale survey data
Source: Ecol Evol. 2022 Oct 1;12(10):e9328. doi: 10.1002/ece3.9328 (PMC9526027; doi:10.1002/ece3.9328)

**Supporting Information**

**Section 1: Choosing a transformation for the distance covariate**

The relationship between distance-to-range and occupancy probability is not expected to be logit-linear. In particular, we expected *a priori* that the occupancy probability in the core range of wide-ranged species would asymptote at a value less than one. We recognized *a priori* that by selecting a transform that mapped strongly negative (i.e. in-range) distances to the vicinity of zero, we would yield a model where the pooling of species-specific intercepts shared information about the core of the range for all species. Thus we initially considered transformations of the form $e^{x}$, where $x$ is the distance-to-range and the measurement unit for $x$ is freely chosen by the researcher. However, these transforms failed to approximately linearize the logit-proportion of points with at least one detection (aggregated across all species).

We then experimented with transforms of the form $e^{sgn(x)*abs({x)}^{n}}$, where $sgn$ is the sign function, $abs$ is the absolute value function, and the exponent $n$ is freely chosen by the researcher. This extra degree of freedom allowed us to approximately linearize logit-proportions choosing $n$ to be near 0.7, but at the cost of transforming the largest positive (out-of-range) distances to extremely large values. As a consequence, this transform resulted in an over-abundance of extremely influential points during model fitting, which made model comparison via approximate leave-one-out cross-validation using pareto-smoothed importance sampling prohibitive. Moreover, we realized after the fact that this transformation requires the estimation of relationships that are *a priori* implausible since in some years (but not in 2018) the breeding bird survey records a species thousands of kilometers out-of-range.

Thus, we finally settled on an inverse-logit transformation of distance, with distance expressed in units of 200 km for the BBS data and units of 14.92 km in the West Andes data (this unit is not round because when we chose it we were working with the scaled raw distances, i.e. the distances divided by their standard deviation). Again these are freely chosen values to approximately linearize the logit-proportions of points with at least one detection, aggregated across all species.

**Section 2: Manual range-map updates in the West Andes avifauna**

Our field data exposed only two errors of omission in the biogeographic clipping that we performed around the range maps of Ayerbe Quiñones (2018):

*Entomodestes coracinus* -- We detected this species on the east slope of the West Andes in a region where Ayerbe Quiñones maps this species as being (barely) restricted to the west slope (thereby triggering biogeographic clipping at the crest of the West Andes). Our records coincide with numerous additional east-slope records, and we manually added polygons around known clusters of east-slope records (eBird 2021).

*Thlypopsis superciliaris* -- We detected this species at multiple locations. Ayerbe Quiñones omits this species from the northern part of the West Andes in Colombia, despite its well documented occurrence in the area. We manually added a polygon around a well-known cluster of records in the northern West Andes (eBird 2021).

We then recomputed the distance-from-range and reimplemented biogeographic clipping based on the updated range maps.

**Section 3: Priors for the bMSOM for the West Andes avifauna**

Vague priors cause problems in occupancy models for modest-sized datasets as they induce pushforward densities on the probability scale that concentrate near probabilities of zero and one (Lele 2015, Northrup & Gerber 2018). We specify principled priors elicited as described below. To ensure that covariate relationships are due to the data and not to our prior model we use zero-centered normal distributions for all coefficients, and we chose weakly informative prior scale parameters to understate the certainty of our prior knowledge while simultaneously avoiding outlandishly concentrated pushforward densities near zero and especially near one (occupancy probabilities may well approach zero, as in the case of strict forest specialists at pasture points, but should rarely approach one). Prior elicitation relied on JBS’s experience conducting bird surveys in Peru (Socolar et al 2019), previous trait-based modeling of neotropical bird declines (Socolar & Wilcove 2019), and previous spatially explicit mapping of neotropical bird territories (Terborgh et al 1990).

To formulate consistently interpretable priors, we standardized most covariates. However, for species-standardized elevation we divided by the standard deviation without subtracting the mean, and for the geographic range covariate we performed no additional transformation beyond what is described in the main text. This ensures that the intercept in our model corresponds to occupancy in the core of a species range. To ensure that pushforward densities are not sensitive to the arbitrary choice of reference category for dummy-coded binary predictors, we code all binary predictors as -1/1 rather than 0/1 (Agresti 2018).

*Occupancy: intercept*

We entertained a non-zero-centered prior for the intercept because zero-centered priors yielded unreasonable pushforward densities on the occupancy probability. Two independent lines of evidence imply that the intercept is substantially less than zero in our model. First, the average occupancy probability, even in the elevational and geographic core of a species’ range, is expected to be substantially less than 0.5. Less than half of the regional lowland avifauna occurs on a 97-hectare bird census plot in Peru, the best-characterized neotropical forest bird community, and within the plot only about half of species occur at any given point (Terborgh et al 1990, Walker et al 2006). This suggests an average occupancy probability of roughly 0.25. Second, we expect a substantial excess of probabilities near zero (even in the core of species’ ranges), corresponding to species-point pairs that are mismatched for habitat. However, we expect a much smaller excess of probabilities near one (spatial effects might induce some excess of probabilities near one for species with very high spatial autocorrelations, but this excess should be much smaller than the excess of near-zero probabilities). Such asymmetry is achievable in the prior pushforward density only via an intercept that is not zero-centered.

If the average occupancy probability is roughly 0.25 in our sample, then by Jensen’s inequality the average logit-probability is less than logit(0.25) = -1. If the variance in the logit-occupancy is large (due to covariate relationships and/or random effects), then the average logit-occupancy will be much less than -1. We were *a priori* certain that the variance in logit-occupancy probabilities is large, but highly uncertain about its magnitude. After defining our priors on the remaining covariates (see below), we examined how our prior choice for the intercept impacts the pushforward density for the occupancy probability of a “typical” species with covariate values near one standard deviation for every predictor. We initially experimented with a prior of Normal(-3,1) as a prior that 1) allows low values for the intercept; 2) allows values up to about -1, which is an upper bound on the plausible intercept values; and 3) results in an acceptable (rather than outrageously large) excess of probabilities near one.

However, model fitting revealed prior-data conflict, with estimates in the range of -7 to -5. Therefore, we implemented a prior of Normal(-7, 2.5) as a prior that 1) allows for extremely low values for the intercept; and 2) allows values up to about -1 without placing excess tail probability on values much greater than -1, thereby remaining consistent with the domain expertise that we had elicited previously.

*Occupancy: range effects*

In our model, two covariates (relative-elevation-squared and distance-to-range) determine whether a species is in-range or out-of-range, and we expect large effect sizes for the associated coefficients. In the context of a large effect size for relative-elevation-squared, we also entertain large effect sizes for the linear relative-elevation term or the interactions of the linear and squared relative-elevation terms with lowland occurrence. We expect these parameters to be strongly identified in the model (and confirm *a posteriori*  that they are indeed strongly identified), mitigating the problems associated with weak priors. Furthermore, these coefficients have no influence on the pushforward distributions for species in the core of their geographic and elevational range. Therefore, we use very weak Normal(0,5) priors for these five coefficients.

*Occupancy: pasture effect*

We expected that the change in point-scale species richness between forest and pasture should be on average smaller than 10-fold. In the limit of low occupancy probability, this ratio corresponds to a change in logit-occupancy of about 2.3, but the corresponding effect size is larger under progressively higher baseline occupancy probabilities. Under the very high baseline occupancy probability of 0.8, this ratio corresponds to a change in logit-occupancy of roughly 4, corresponding to an effect size of 2 under our effects-coding of the interaction. We use a prior of Normal(0,1).

*Occupancy: trait effects*

Species traits might influence overall occupancy in our dataset if they are predictive of restriction to undersampled habitats, but any such effects are almost certainly not larger than the influence of traits on abundance differences between forest and pasture (see below). Aside from these effects, evidence from Peru suggests that traits are only weakly related to occupancy (Terborgh et al 1990, Russo et al 2003). Therefore, we used Normal(0, 1) priors for the main effects of traits involving habitat associations and biogeographic patterns, and Normal(0, 0.5) priors on coefficients involving body-mass or diet (see below).

*Occupancy: trait-by-pasture interactions*

Previous work in Peru suggests that the most important species traits, such as forest specialization, alter the log abundance ratio between forest and pasture by no more than about 4 (Socolar & Wilcove 2019). In the limit of low occupancy probability, this log-ratio corresponds to a change in logit-occupancy of 4, but the corresponding effect size is larger under progressively higher baseline occupancy probabilities. Under the very high baseline occupancy probability of 0.8 in preferred habitat, this log-ratio corresponds to a change in logit-occupancy of roughly 7, corresponding to an effect size of 1.75 under our effects-coding of the interaction.

Other traits have universally smaller effects, with traits related to habitat and biogeography consistently showing larger effects than traits related to body mass or diet. Therefore, we used Normal(0,1) priors for traits involving habitat or biogeography, and Normal(0,0.5) priors for traits involving body mass or diet.

*Occupancy: random effect standard deviations*

For terms with taxonomic effects of species and family, we found it easier to reason about the magnitude of the total taxonomic variation (i.e. the combined species- and family-level variation) as opposed to the species- and family-level variation in isolation. Likewise, for terms with spatial effects of both cluster and subregion, we found it easier to reason about the magnitude of the total spatial variation (i.e. the combined cluster- and subregion-level variation). Therefore, we specified these random effects by placing a half-normal prior on the square root of the combined variance, and then additively partitioning the combined variance into the two levels using a multiplicative parameter p (and its complement, one minus p), on which we placed a Uniform(0,1) prior.

To choose a prior for the standard deviation of the combined species- and family-level random intercepts, we examined our expectations for the range of typical occupancy probabilities for different species in preferred habitat (forest/pasture) in the core of their range. We felt that an appropriate distribution should be capable of covering probabilities whose logits span a range of at least 9 (e.g. probabilities as low as 1e-3 for species like *Geotrygon violacea* and *Myrmornis torquata* and as high as 0.9 for species like *Tangara chilensis* and *Zonotrichia capensis*). Thus, we chose a standard deviation of 2 for the half-normal prior on the square root of the taxonomic variance.

To choose a prior for the standard deviation of the combined species- and family-level random slopes for pasture, we considered the residual variation in responses to pasture that we would expect to find within a cohort of species with similar trait values. For example, the cohort of species that are classified as forest-present but not forest-specialist includes *Tyrannus melancholicus*, which we expected would be abundant in pasture (occupancy probabilities in excess of 0.5) and nearly absent from forests (occupancy probabilities well below 0.01), and also species like *Coeligena helianthea*, which we expected would be substantially commoner in forest than in pasture. Thus, we again felt that the random effect distribution should be capable of covering probabilities whose logits span a range of about 9, which is comfortably covered by a half-normal prior with standard deviation 1 (due to the effects coding of forest vs. pasture).

Our prior expectations were weakest for the standard deviation of the combined cluster- and subregion-level random intercepts. We felt that species modeled as present with moderate probability might be locally absent with very high probability due to the influence of unmeasured habitat features (e.g. edaphic variables). Likewise, we felt that species modeled as absent with very high probability might occur in a highly spatially correlated fashion, with high probabilities of point-level occupancy conditional on occupancy of a cluster. Therefore, we selected a very weak half-normal(0, 3) prior on the square root of the spatial variance.

We chose weak Normal(0,2) priors for the species-level slopes for relative-elevation and squared-relative-elevation. We chose these weak priors in the context of our expectation for large effect sizes in these terms and the fact that these terms do not influence the pushforward density for occupancy probabilities in the core of species ranges (see *Occupancy parameters: range effects* above).

*Detection: intercept*

Based on direct experience surveying neotropical birds on spot-mapped plots where the territories were known, we *a priori* expected average detection probabilities to be much less than 0.5, since even at the height of the dawn chorus a ten-minute point-count always detects less than half of the species present. At the same time, we felt that average values below 0.05 are *a priori* implausible (but note that due to Jensen’s inequality this lower bound estimate might correspond to an average logit substantially less than logit(0.05) = -2.9). By examining the influence of the intercept on the average of the pushforward density for the detection probability based on the full prior uncertainty for the remaining detection parameters, we found that an average probability of 0.05 is obtained when the intercept is near -6, but this estimate is expected to be substantially too low because our priors intentionally overstate the prior uncertainty in the remaining parameters. Therefore, we use a prior of Normal(-3,1) on the intercept.

*Detection: pasture effect*

We expected the effect of pasture to combine two countervailing effects. On one hand, sight lines are longer and sound propagates better in pasture than in forest. On the other hand, birds with territories that span both forest and pasture might enter the pasture portion of their territories (e.g. to forage in isolated trees; Boesing et al 2021) only infrequently. We thought that either of these effects might be large, potentially causing average detection probabilities to swing across most of their *a priori* plausible range. Due to our effects coding, we use a prior of Normal(0, 0.75) to cover such a swing.

*Detection: time and elevation effects*

Based on direct experience surveying neotropical birds, we expected detection to decay substantially though the morning in the lowlands, but not necessarily in the highlands, with effect sizes as large as a roughly ten-fold change in probability, corresponding with logit-scale changes on the order of 3 over covariate values of +/- 2-sigma. To understate our prior certainty, we used priors of Normal(0, 0.5) for the effects of time, median elevation, and the interaction between the two.

*Detection: observer effects*

Because all observers recorded sound continuously during point-counts and consulted *inter se* to identify unknown sounds, we expected observer effects to be small. We used a prior of Normal(0, 0.25) for observer effects.

*Detection: trait effects*

Previous work (in a boreal system) suggests that trait-based covariates have modest influences avian on detection probabilities (Sólymos et al 2017). We used a prior of Normal(0, 0.5) for all traits except migratory status. Because overwintering migrants are expected to be potentially much less vocal than other taxa, we allow for a larger effect size of migratory behavior, with a prior of Normal(0, 1).

*Detection: random effect standard deviations*

As for occupancy, we thought about random effect variances for detection in terms of a combined “taxonomic variance” that subsumes the species- and family-level variances.

For the intercept, we thought that detection might vary across taxa over a range of approximately 0.001 (e.g. *Neomorphus, Harpia, Nothocrax*) to 0.8, or approximately 9 units of logit-probability. We chose a half-normal prior with standard deviation 2 for the square root of the taxonomic variance.

We expected the effect of pasture to combine two countervailing effects whose importance might vary by species (see *Detection: pasture effect* above). Therefore, we thought that the random-effect variation might potentially exceed our prior uncertainty in the mean effect size, and so we chose a half-normal prior with standard deviation 1 for the square root of the taxonomic variance.

For species-specific variation in time-of-day relationships, we expected substantial variation between species that regularly join the dawn chorus versus species that rarely sing but are often detected in mixed flocks that form later in the morning. Therefore, we use a half-normal prior with standard deviation 1 on the standard deviation of the species effect variance.

**Section 4: The saga of the data-augmented model**

We attempted to fit the data-augmented model under the same priors as the bMSOM for all shared parameters, replacing the species-standardized elevations with scaled raw elevations and placing a uniform prior on $\omega$, the probability that a given species is included in the metacommunity. However, model fitting proved challenging. During the early phase of warmup all MCMC chains entered a region of the posterior where they consistently saturated the treedepth and failed to mix quickly. As warmup proceeded, the chains adapted to the geometry of this region of the posterior but failed to explore the bulk of the posterior. Every chain entered a region where the standard deviation of the species-specific random intercept for either occupancy or detection approached zero (e.g. values less than $10^{-10}$) and then slowly recovered. Yet this recovery was so glacially slow that it quickly became clear that we could not achieve correct inference using our available computational resources except by modifying our approach.

Fortunately, all chains agreed in estimating extremely high values of $\omega$, essentially equal to one. We noted that if we fix $\omega$ to 1, the data-augmented MSOM becomes equivalent to the traditional MSOM fit to the augmented dataset. On the hypothesis that $\omega$ would remain near one at convergence in the data-augmented model, we fit the traditional MSOM to the augmented data to learn the approximate location and scale of the posterior. The traditional model ran much faster. Because our main purpose was to obtain useful estimates for the inverse metric and for initial values in the data-augmented model, we ran four chains until one chain completed its 1000 warmup iterations, at which point we terminated the computation.

We then extracted the inverse metric from the chain that finished warmup as well as the final iteration from all four chains, and we again attempted to fit the data-augmented MSOM, this time initializing the inverse metric using the diagonal metric extracted from the traditional MSOM and initializing the chains at the final iterations from each of the four chains from the traditional MSOM. We initialized the diagonal inverse metric entry for $\omega$ to Stan’s default value of one, and the intercept for $\omega$ at 0.990. To avoid bad early updates to the inverse metric, we also specified that the initial adaptation window for computing the inverse metric should run for 50 iterations rather than the default 25. To avoid unnecessarily long integration times after warmup we specified that the term buffer should run for 100 iterations rather than the default 50. This second attempt at fitting data-augmented MSOM immediately showed much better mixing behavior than our initial attempt, but still took roughly a week per chain to run. One of the four chains was slightly less successful in its adaptation and adapted to a step-size that required an increase in treedepth from 7 to 8 and therefore twice the computation time. We terminated this chain. At the end of model fitting, r-hat values for some parameters (computed over the three chains that were retained) were problematic (as high as 1.16). However, all three chains again agreed that $\omega$ was very high (95% credible interval 0.9977–1.0000, r-hat = 1.01). The chains also agreed that the occupancy intercept was extremely low (95% credible interval -13.8 – -16.8, r-hat = 1.04), which is low enough to substantially conflict with our already-low prior of Normal(-7.5, 2.5).

Thus, in a final step, we switched back to the traditional MSOM, confident that the data-augmented model truly yielded $\omega$ values near unity, and we replaced the Normal(-7.5, 2.5) prior on the occupancy intercept with a logistic prior (i.e. a flat prior on the probability scale), which decays marginally slower than Normal(-7.5, 2.5) in the region of -13.8 – -16.8. To avoid spending excessive computational resources on warmup, we terminated this model after one chain finished warmup and then ran four new chains with no warmup using initial values taken from the final state of the previous chains, and inverse metric and step size taken from the chain that finished warmup. Even still, parts of this model did not converge, especially the detection intercept (rhat = 1.14) and species-specific parameters related to detection (rhat as high as 1.16). The lack of convergence likely reflects the inherent misspecification of the data-augmented model for this system. Nevertheless, all hyperparameters in the occupancy sub-model were somewhat better behaved (maximum r-hat = 1.04) and so we used this final model as the basis for tentative inference on patterns of species richness.

**Supplementary references**

Agresti, Alan. 2018. Introduction to Categorical Data Analysis. 3rd ed. John Wiley & Sons.

Ayerbe Quiñones, F. 2018. Guia Ilustrada de la Avifauna Colombiana. WCS Colombia, Bogotá.

Boesing, A.L. *et al* 2021. Conservation implications of a limited avian cross-habitat spillover in pasture lands. Biological Conservation 253:108898.

eBird. 2021. eBird: An online database of bird distribution and abundance [web application]. eBird, Cornell Lab of Ornithology, Ithaca, New York. Available: http://www.ebird.org.

Lele, S. 2015. Is non-informative Bayesian analysis appropriate for wildlife management: survival of San Joaquin Kit Fox and declines in amphibian populations. arXiv:1502.00483

Northrup, J.M. & B.D. Gerber. 2018. A comment on priors for Bayesian occupancy models. PLoS ONE 13: e0192819.

Parker, T.E. *et al*. 1996. Ecological and Distributional Databases for Neotropical Birds. Chicago, IL: Chicago University Press.

Russo, S.E. *et al.* 2003. Size-Abundance Relationships in an Amazonian Bird Community: Implications for the Energetic Equivalence Rule. American Naturalist 161: 267-283.

Socolar, J.B., E.H. Valderrama Sandoval, & D.S. Wilcove. 2019. Overlooked biodiversity loss in tropical smallholder agriculture. Conservation Biology 33: 1338-1349.

Socolar, J.B. & D.S. Wilcove. 2019. Forest-type specialization strongly predicts avian responses to tropical agriculture. Proceedings of the Royal Society B 286: 20191724.

Sólymos, P et al. 2017. Phylogeny and species traits predict bird detectability. *Ecography* 41: 1595–1603.

Terborgh, J. W. *et al* (1990). Structure and organization of an Amazonian forest bird community. Ecological Monographs. 60: 213-238.

Walker, B. *et al*. 2006. Birds of the Manu Biosphere Reserve. Fieldiana Zoology 110: 23-49.

Wilman, H. *et al*. 2014. EltonTraits 1.0: Species-level foraging attributes of the world's birds and mammals. Ecology 95: 2027.

**Supplementary Tables**

**Table S1** Model covariates and grouping terms for the West Andes

| **class** | **name** | **interpretation** |
| --- | --- | --- |
| grouping variables for random effects | species | taxonomic species |
|  | family | taxonomic family |
|  | species:cluster | species by sampling cluster |
|  | species:subregion | species by sampling subregion (20 km scale) |
|  | species:observer | Species by observer |
| covariates for both occupancy and detection | habitat | forest or pasture (coded -1/1) |
|  | Mass | body mass (standardized) |
|  | elevMedian | median of max/min elevation reported by Ayerbe (standardized) |
|  | migratory | migratory or non-migratory (coded -1/1) |
|  | dietCarn | carnivorous or non-carnivorous^1^ (coded -1/1) |
| covariates for occupancy only | elev | species-standardized elevation |
|  | elev2 | Squared species-standardized elevation |
|  | lowland | is the minimum elevation reported by Ayerbe in the lowlands? coded -1/1 |
|  | elev:lowland | interaction of elev and lowland |
|  | elev2:lowland | interaction of elev2 and lowland |
|  | mtnBarrier | does the species occur on both sides of the East Andes in Colombia? (coded -1/1) |
|  | valBarrier | does the species occur both east of the Magdalena Valley and west of the Cauca Valley in Colombia? (coded -1/1) |
|  | elevBreadth | difference of max/min elevation reported by Ayerbe (standardized) |
|  | forestPresent | does the species occur in forest habitat?^2^ (coded -1/1) |
|  | forestSpecialist | does the species occur exclusively in forest habitat?^2^ (coded -1/1) |
|  | tfSpecialist | does the species occur exclusively in non-flooded forest habitat?^2^ (coded -1/1) |
|  | dryForestPresent | does the species occur in dry forest?^2^ (coded -1/1) |
|  | floodDrySpecialist | is the species restricted to dry forest and/or floodplain forest?^2^ (coded -1/1) |
|  | aridPresent | does the species occur in deserts?^2^ (coded -1/1) |
|  | elevMedian:forestPresent | interaction of elevMedian with forestPresent |
|  | elevMedian:forestSpecialist | Interaction of elevMedian with forestSpecialist |
|  | dietInvert | insectivorous or non-insectivorous^1^ (coded -1/1) |
|  | dietFruitNect | nectivorous or frugivorous^1^ (coded -1/1) |
|  | dietGran | granivorous or non-granivorous^1^ (coded -1/1) |
|  | habitat interactions | interactions of habitat with all other occupancy covariates except distanceToRange |
|  | distanceToRange | inverse logit of signed distance to range margin |
| covariates for detection only | time | hours post-sunrise (standardized) |
|  | time:elevMedian | Interaction of time and elevMedian |

1 Based on Wilman et al 2014. Wilman et al classify every bird to exactly one of five dietary categories; we take “omnivore” as the reference category and include covariates for all remaining categories.

2 Based on Parker et al 1996.

**Table S2** Parameter estimates for hyperparameters and fixed effects in the West Andes. (Continued on next page).

| **class** | **name** | **grouped by** | **mean** | **lower CI**^1^ | **upper CI**^1^ |
| --- | --- | --- | --- | --- | --- |
| fixed effects on occupancy | intercept |  | -7.27 | -9.59 | -4.99 |
|  | elev |  | -3.67 | -5.10 | -2.30 |
|  | elev2 |  | -5.44 | -6.91 | -4.09 |
|  | habitat |  | 1.49 | 0.26 | 2.75 |
|  | distanceToRange |  | -3.50 | -4.83 | -2.24 |
|  | lowland |  | -0.22 | -0.92 | 0.50 |
|  | elev:lowland |  | -4.63 | -6.01 | -3.26 |
|  | elev2:lowland |  | 0.70 | -0.52 | 1.81 |
|  | mtnBarrier |  | 0.28 | -0.35 | 0.91 |
|  | valBarrier |  | -0.21 | -0.96 | 0.55 |
|  | elevMedian |  | -0.70 | -1.71 | 0.29 |
|  | elevBreadth |  | -0.03 | -0.69 | 0.63 |
|  | forestPresent |  | 1.74 | 0.84 | 2.65 |
|  | forestSpecialist |  | 0.81 | 0.16 | 1.46 |
|  | tfSpecialist |  | 0.37 | -0.12 | 0.87 |
|  | dryForestPresent |  | -0.07 | -0.64 | 0.51 |
|  | floodDrySpecialist |  | -0.09 | -1.39 | 1.18 |
|  | aridPresent |  | -0.40 | -1.43 | 0.61 |
|  | migratory |  | -0.93 | -2.19 | 0.39 |
|  | elevMedian:forestPresent |  | 0.60 | -0.37 | 1.58 |
|  | elevMedian:forestSpecialist |  | 0.38 | -0.26 | 1.03 |
|  | mass |  | -0.74 | -1.34 | -0.14 |
|  | dietInvert |  | -0.33 | -0.80 | 0.13 |
|  | dietCarn |  | -0.57 | -1.35 | 0.21 |
|  | dietFruitNect |  | 0.34 | -0.13 | 0.82 |
|  | dietGran |  | -0.06 | -0.67 | 0.55 |
|  | habitat:mtnBarrier |  | -0.24 | -0.65 | 0.15 |
|  | habitat:valBarrier |  | -0.32 | -0.79 | 0.16 |
|  | habitat:elevMedian |  | -0.64 | -1.47 | 0.16 |
|  | habitat:elevBreadth |  | 0.71 | 0.32 | 1.10 |
|  | habitat:forestPresent |  | -0.45 | -1.18 | 0.23 |
|  | habitat:forestSpecialist |  | -2.03 | -2.48 | -1.59 |
|  | habitat:tfSpecialist |  | 0.21 | -0.08 | 0.51 |
|  | habitat:dryForestPresent |  | 0.60 | 0.23 | 0.97 |
|  | habitat:floodDrySpecialist |  | 0.03 | -0.91 | 1.00 |
|  | habitat:aridPresent |  | 0.51 | -0.33 | 1.39 |
|  | habitat:migratory |  | 0.37 | -0.39 | 1.12 |
|  | habitat:elevMedian:forestPresent |  | 0.32 | -0.52 | 1.16 |
|  | habitat:elevMedian:forestSpecialist |  | 0.32 | -0.08 | 0.72 |
|  | habitat:mass |  | -0.12 | -0.45 | 0.21 |
|  | habitat:dietInvert |  | -0.34 | -0.64 | -0.04 |
|  | habitat:dietCarn |  | -0.48 | -1.18 | 0.21 |
|  | habitat:dietFruitNect |  | -0.02 | -0.33 | 0.29 |
|  | habitat:dietGran |  | -.24 | -0.26 | 0.74 |
| occupancy random effect standard deviations | sd_intercept_taxonomic | species, family^2^ | 3.99 | 3.22 | 4.83 |
|  | p_intercept_taxonomic | proportion species^3^ | 0.63 | 0.42 | 0.84 |
|  | sd_intercept_spatial | cluster, subregion^2^ | 2.81 | 2.54 | 3.11 |
|  | p_intercept_spatial | proportion cluster^3^ | 0.63 | 0.56 | 0.70 |
|  | sd_elev | species | 2.85 | 2.21 | 3.47 |
|  | sd_elev2 | species | 2.66 | 2.02 | 3.38 |
|  | sd_habitat_taxonomic | species, family^2^ | 1.57 | 1.26 | 1.90 |
|  | p_habitat_taxonomic | proportion species^3^ |  |  |  |
|  | sd_distanceToRange | species | 3.69 | 1.63 | 5.30 |
| fixed effects on detection | intercept |  | -4.50 | -5.41 | -3.56 |
|  | habitat |  | -0.07 | -0.22 | 0.08 |
|  | time |  | -0.20 | -0.25 | -0.16 |
|  | mass |  | -0.41 | -0.66 | -0.16 |
|  | elevMedian |  | -0.02 | -0.24 | 0.18 |
|  | migratory |  | -1.35 | -1.87 | -0.79 |
|  | dietCarn |  | -0.62 | -1.31 | 0.12 |
|  | time:elevMedian |  | -0.12 | -0.17 | -0.06 |
| detection random effect standard deviations | sd_intercept_taxonomic | species, family^2^ | 1.21 | 1.02 | 1.42 |
|  | p_intercept_taxonomic | proportion species^3^ | 0.85 | 0.66 | 0.98 |
|  | sd_intercept_spObs | species:observer | 0.23 | 0.16 | 0.30 |
|  | sd_habitat_taxonomic | species, family^2^ | 0.70 | 0.59 | 0.83 |
|  | p_habitat_taxonomic | proportion species^3^ | 0.91 | 0.80 | 0.99 |
|  | sd_time | species | 0.20 | 0.15 | 0.24 |

1 Lower and upper bounds of a 95% credible interval (i.e. 0.025 and the 0.975 quantiles of the marginal posterior).

2 This is the square root of the combined variance associated with random effects of species and family (or cluster and subregion).

3 This is the proportion of the combined variance that is associated with species rather than family (or cluster rather than subregion).

**Supplementary Figures**

**
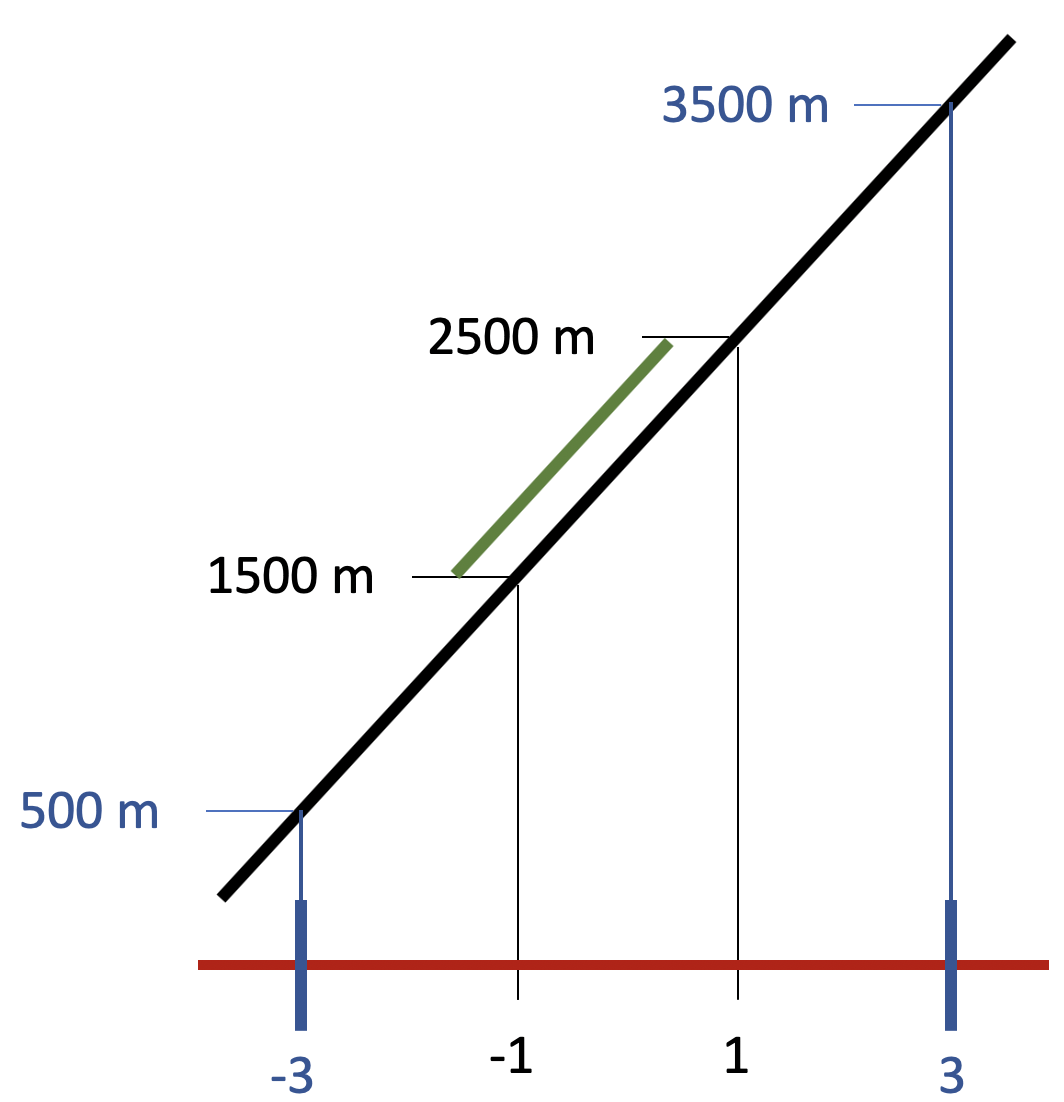
**

**Figure S1** We compute species-standardized elevations for each sampling point and perform biogeographic clipping as follows. We linearly rescale the actual elevation gradient (black line) to a new species-specific gradient (red line) such that the published species-specific minimum and maximum elevations (in this example 1500 m and 2500 m) correspond to -1 and 1. We then perform biogeographic clipping at species-standardized elevations of -3 and 3, in this case corresponding to 500 m and 3500 m.

**Figure S2 (begins on next page)** Predicted occupancy probabilities across the coterminous United States for all 51 modeled warbler species based on the traditional multi-species occupancy model (traditional MSOM), the biogeographic model (bMSOM), and the clipped biogeographic model (clipped bMSOM). The color-scale is equivalent to the scale in figures 2 and 3 of the main text.


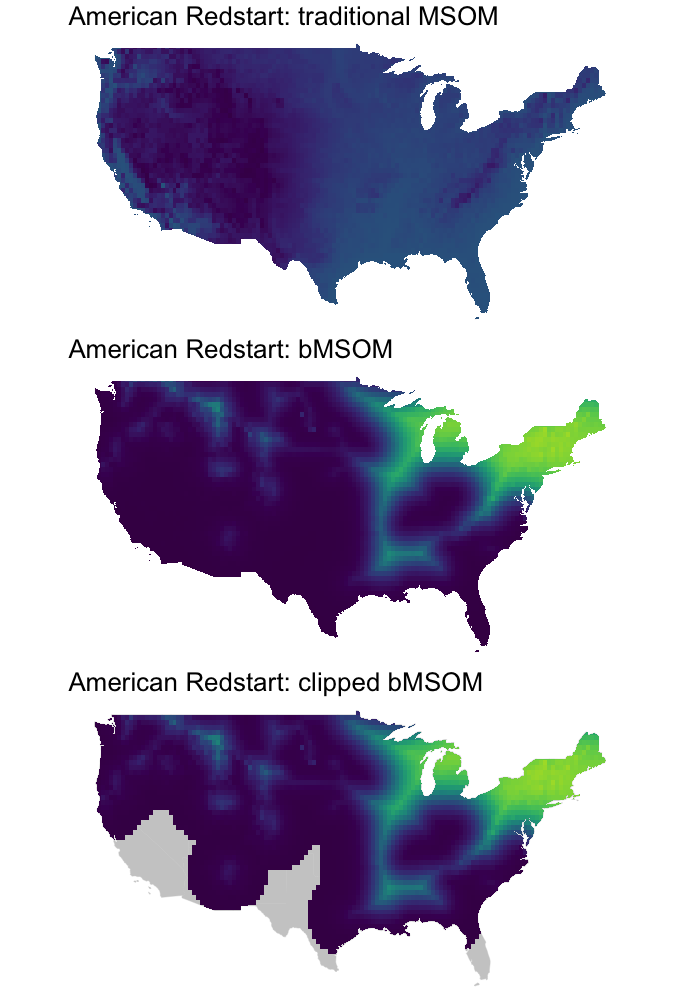

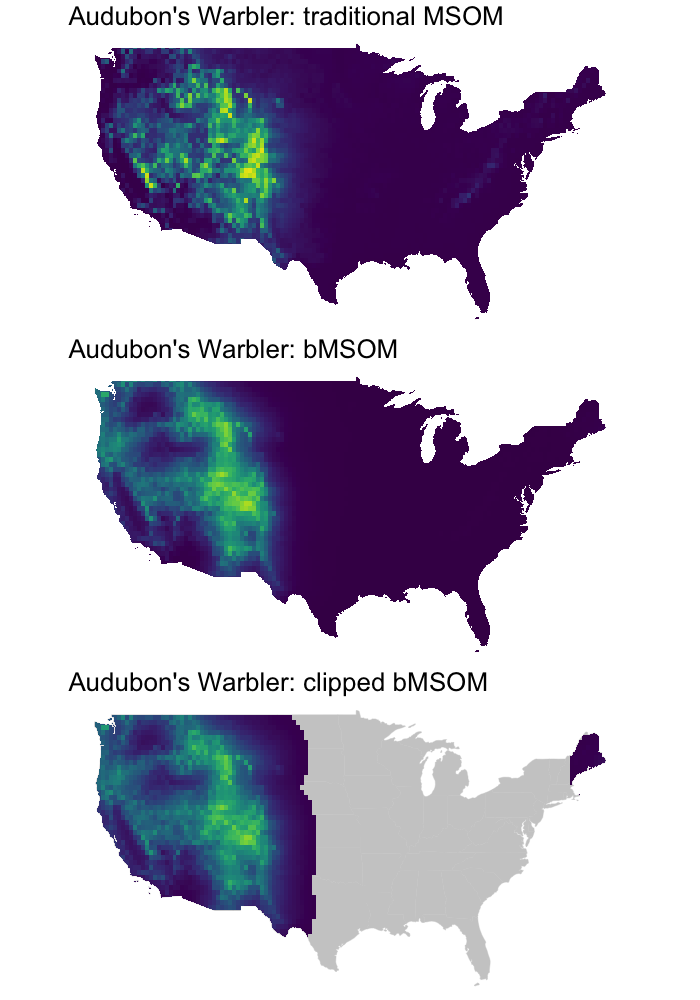


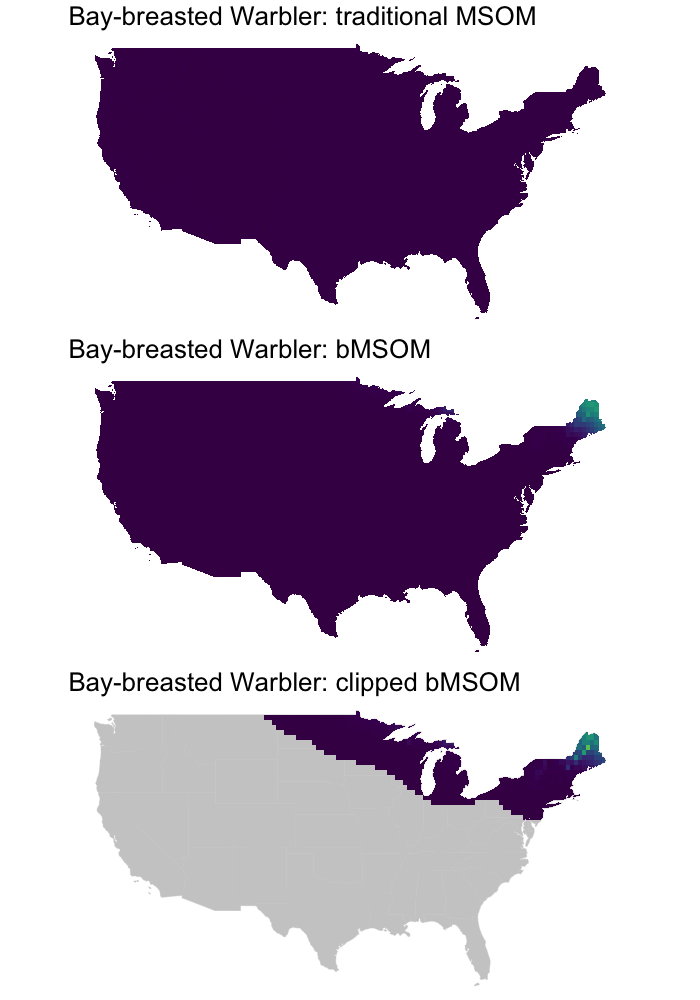

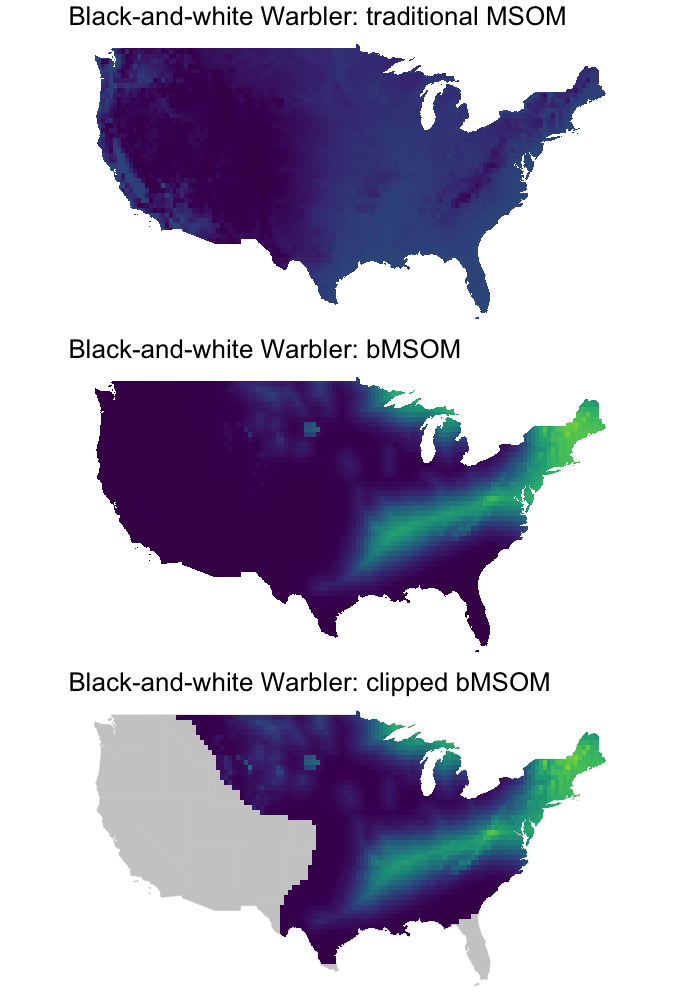


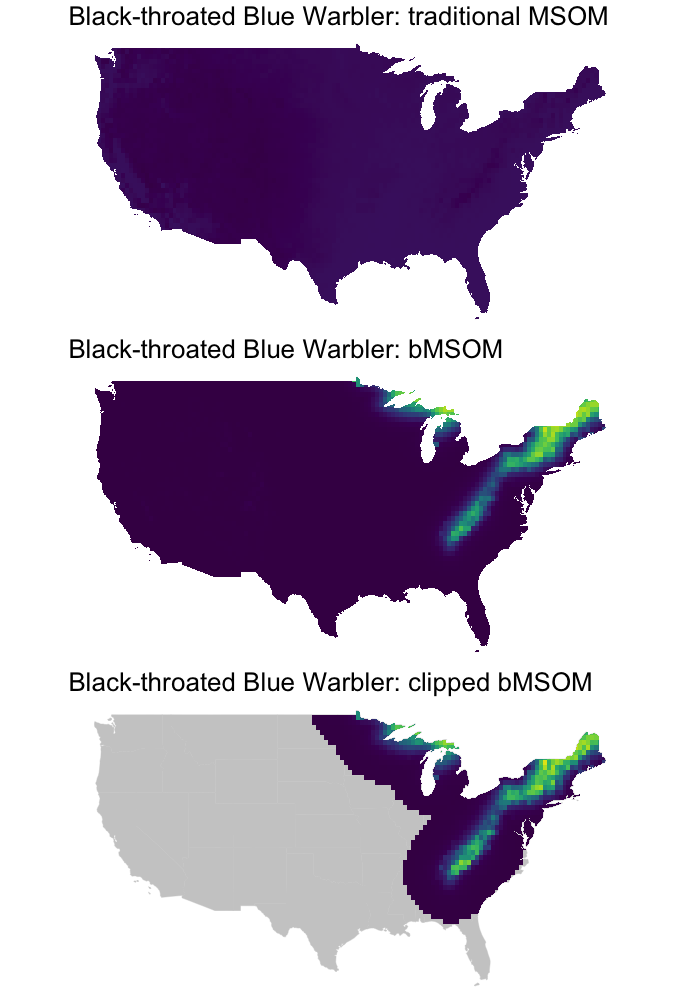

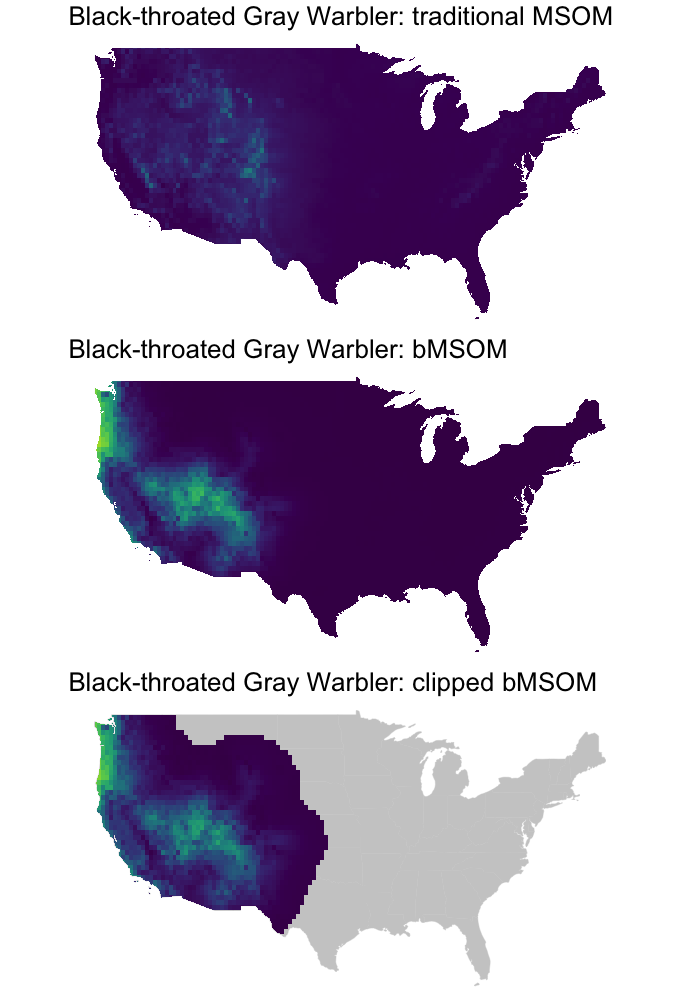


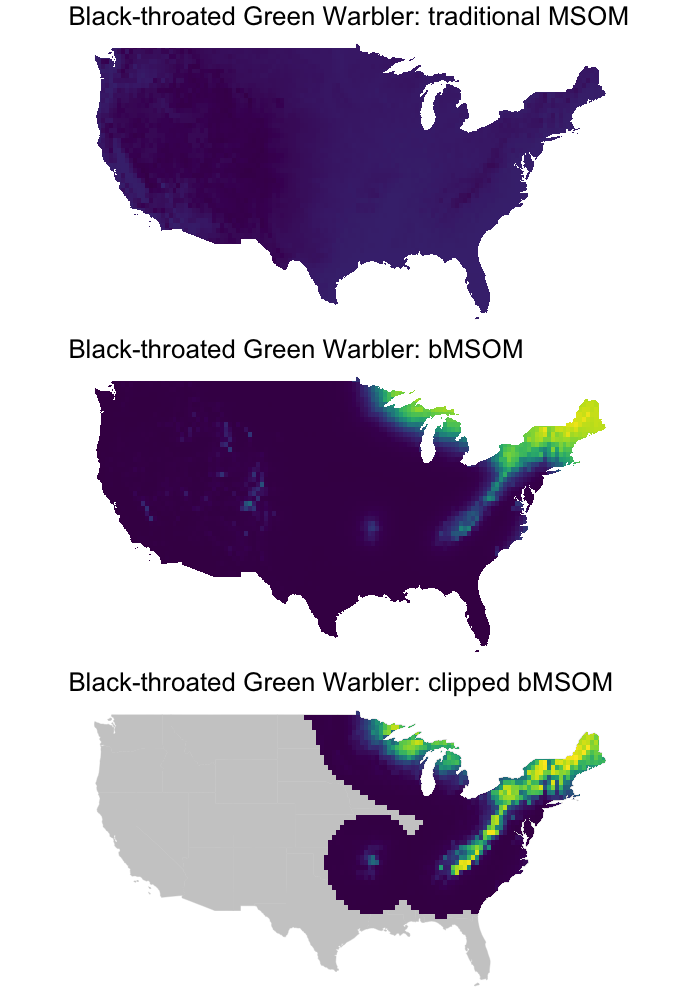

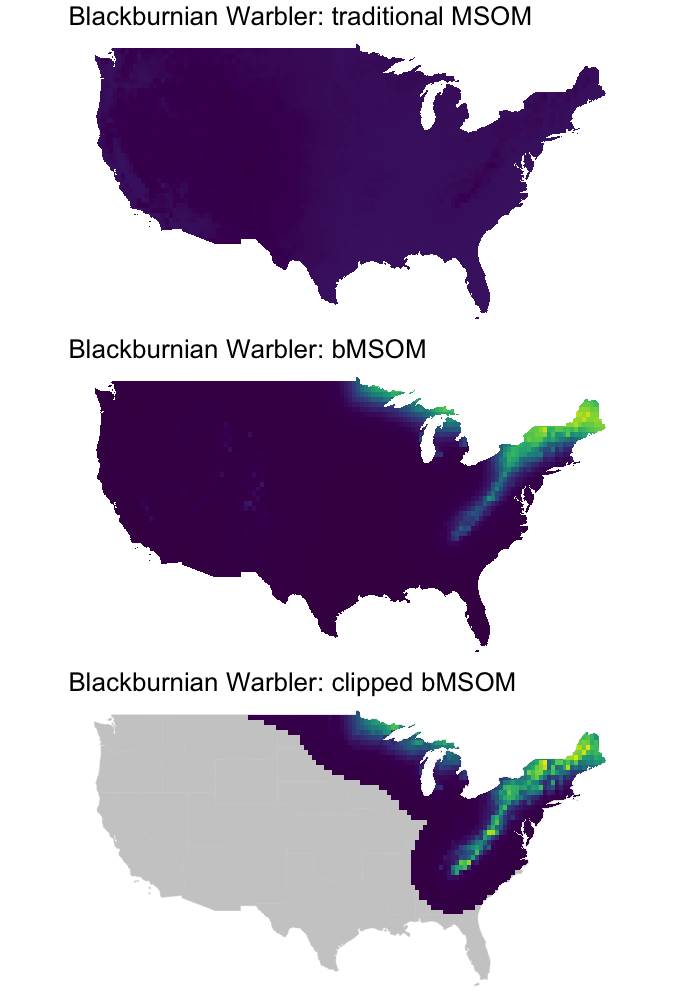


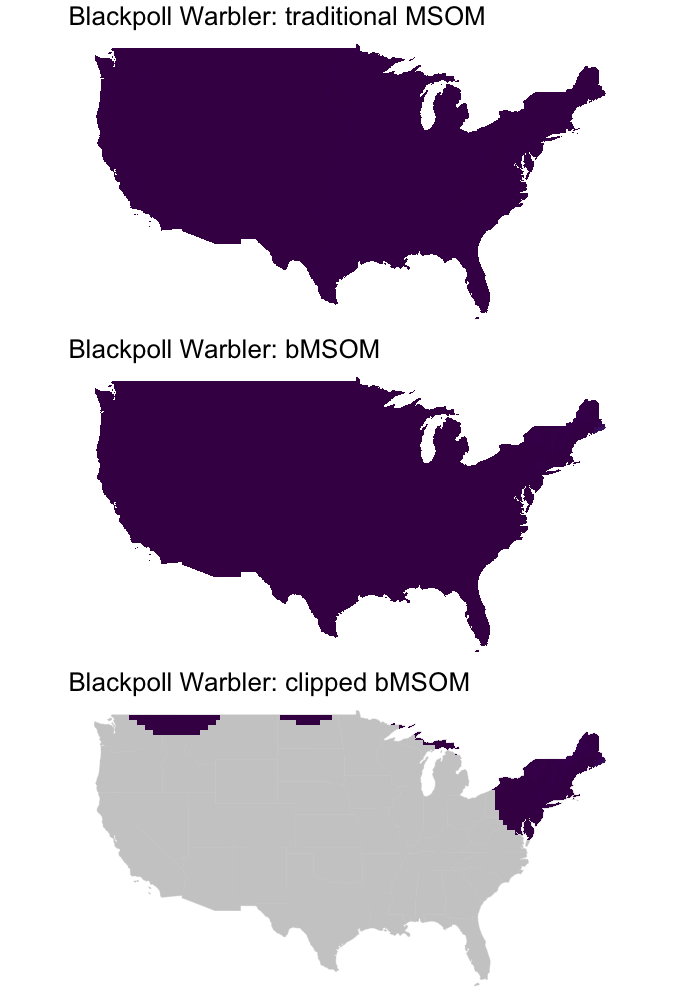

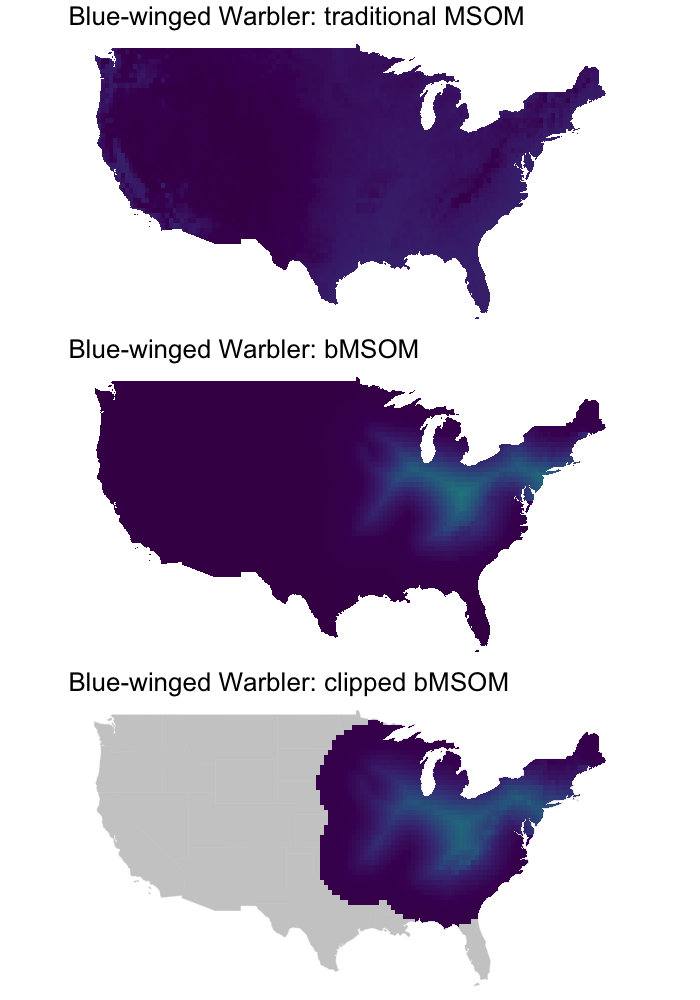


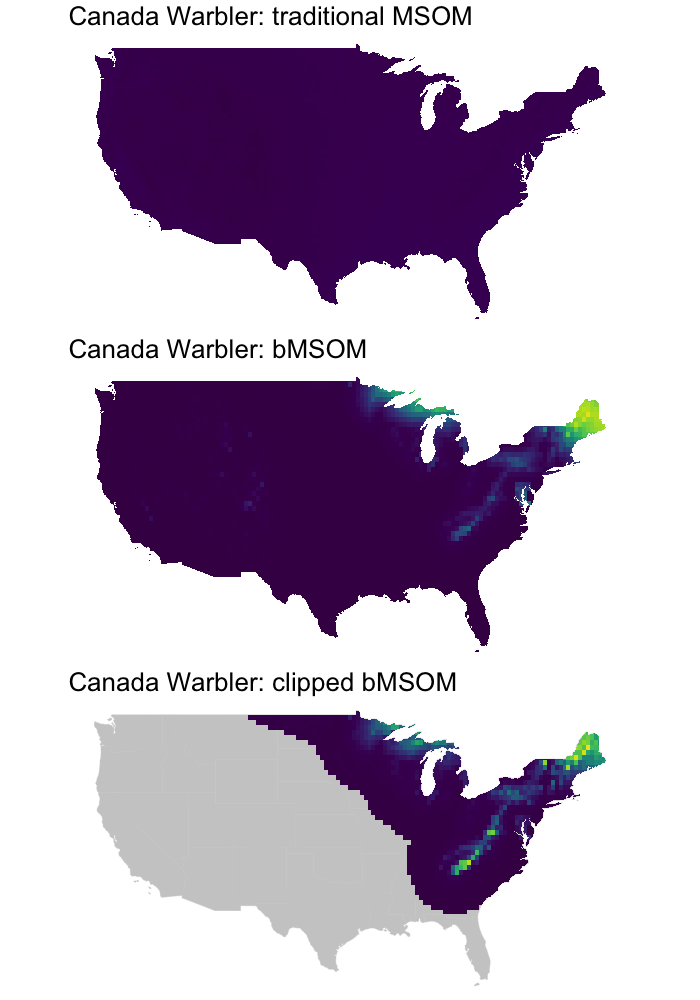

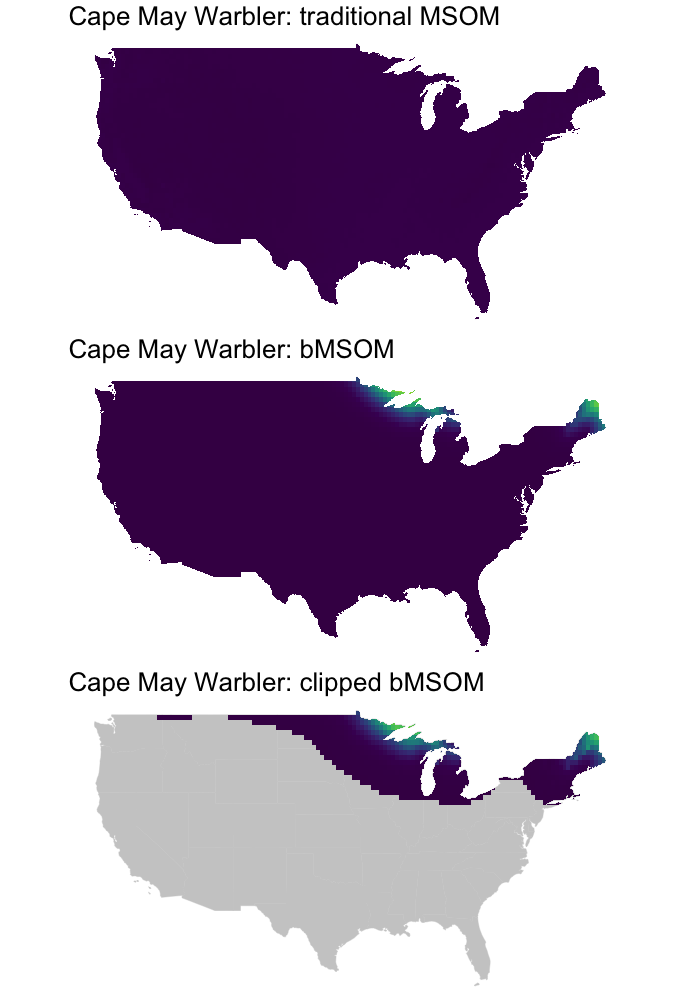


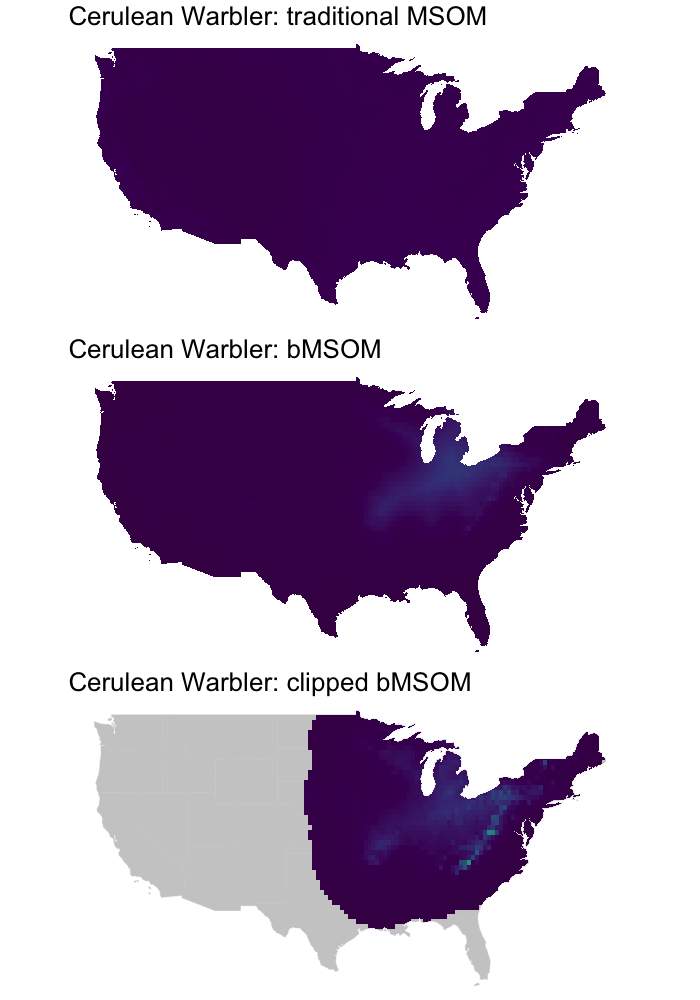

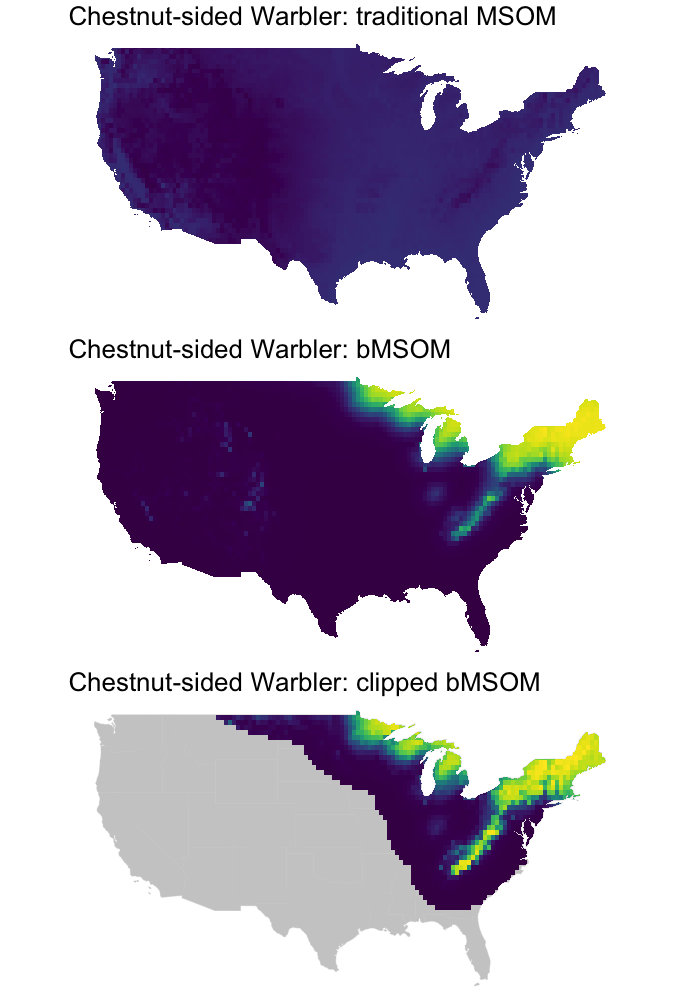


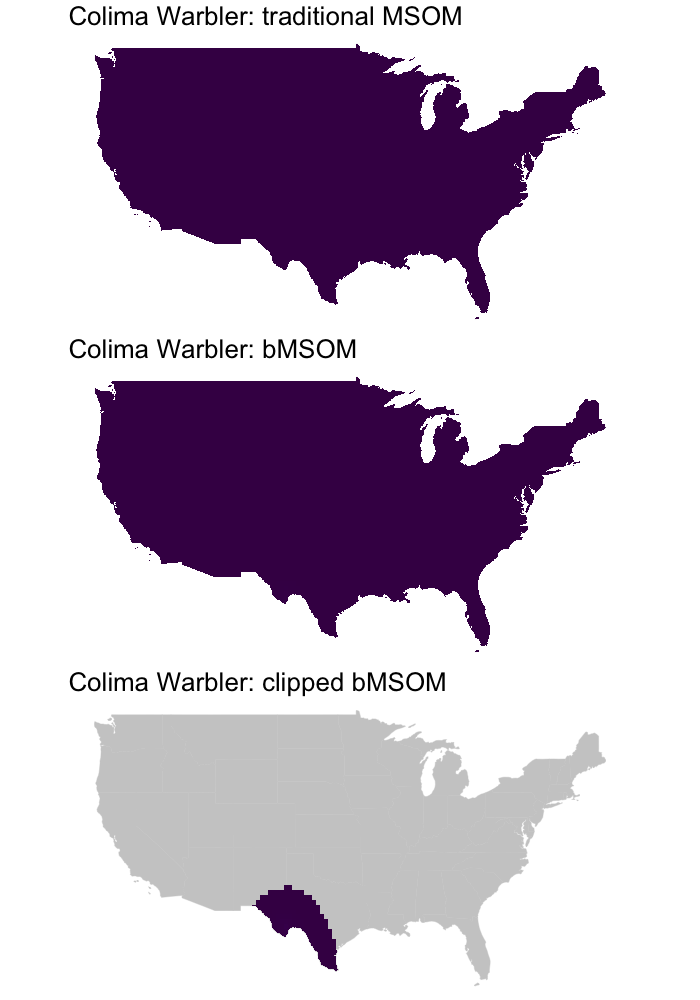

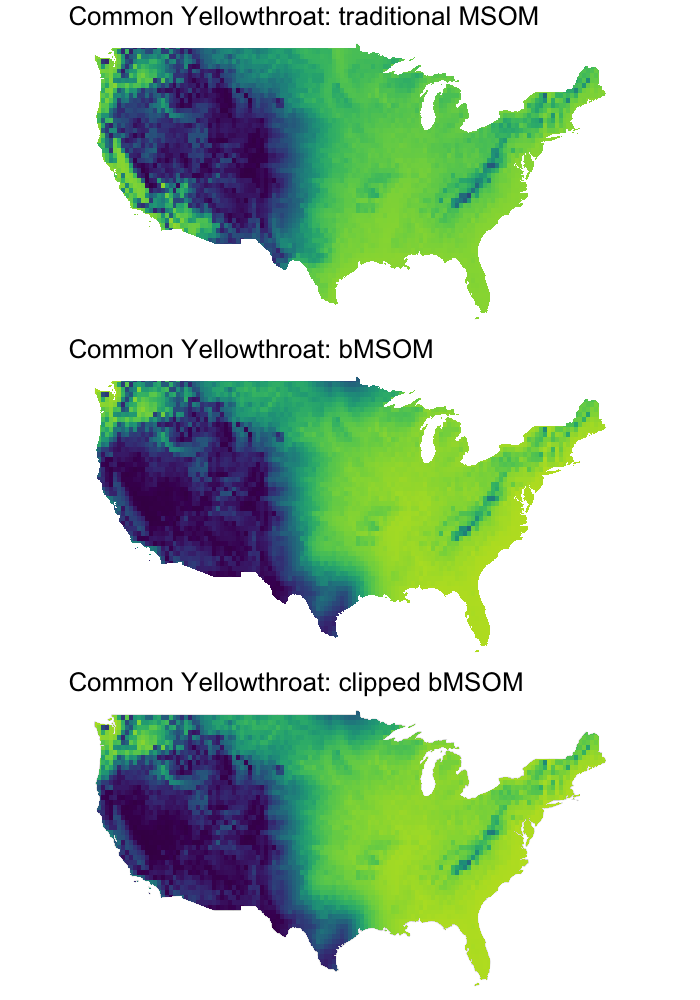


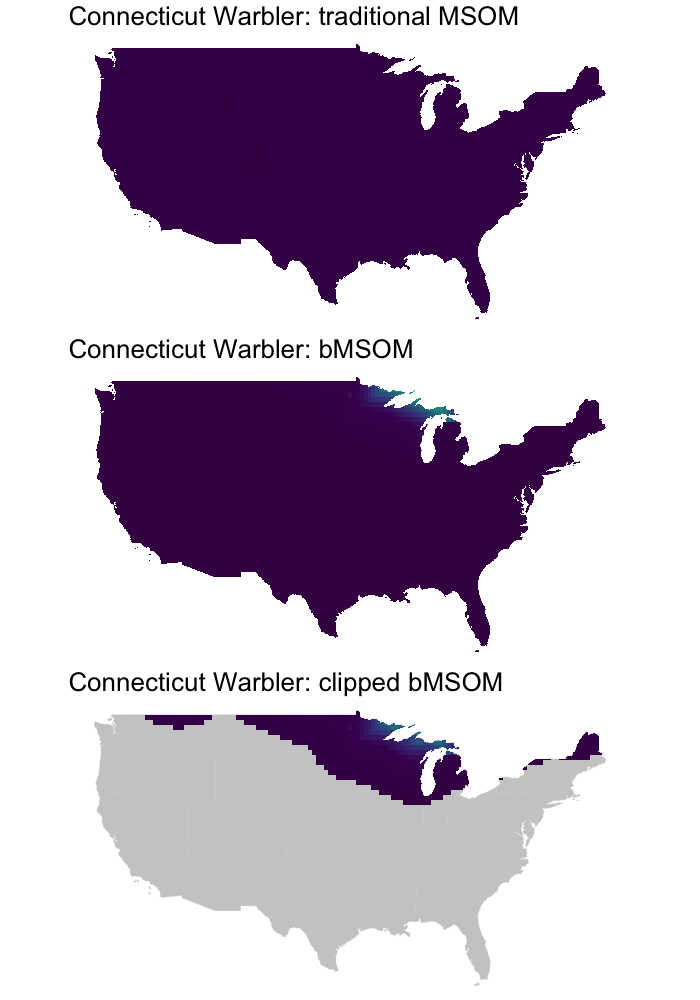

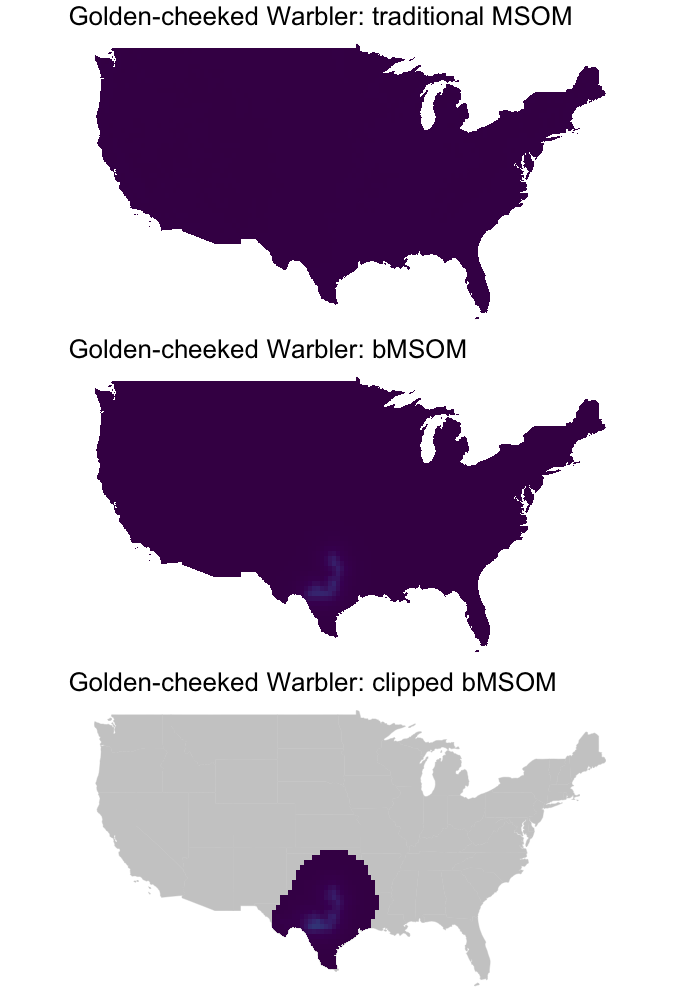


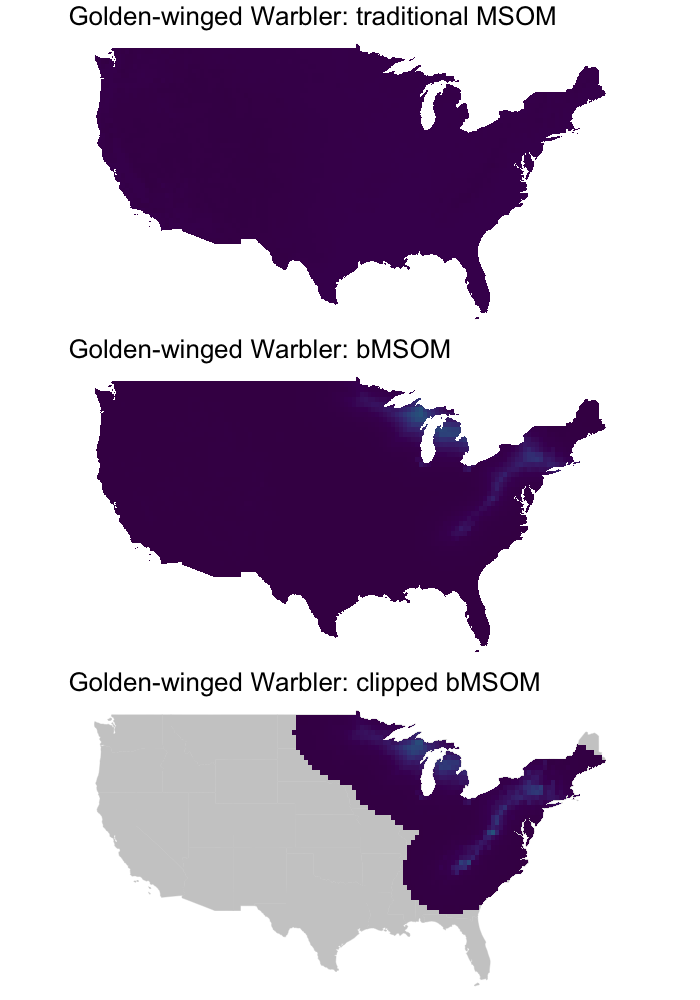

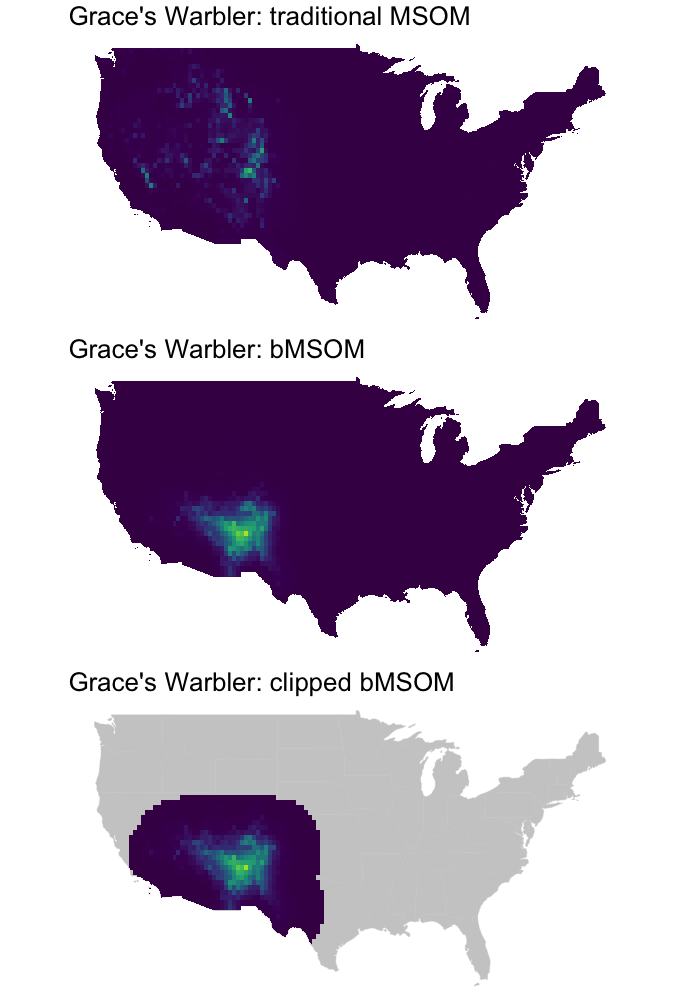


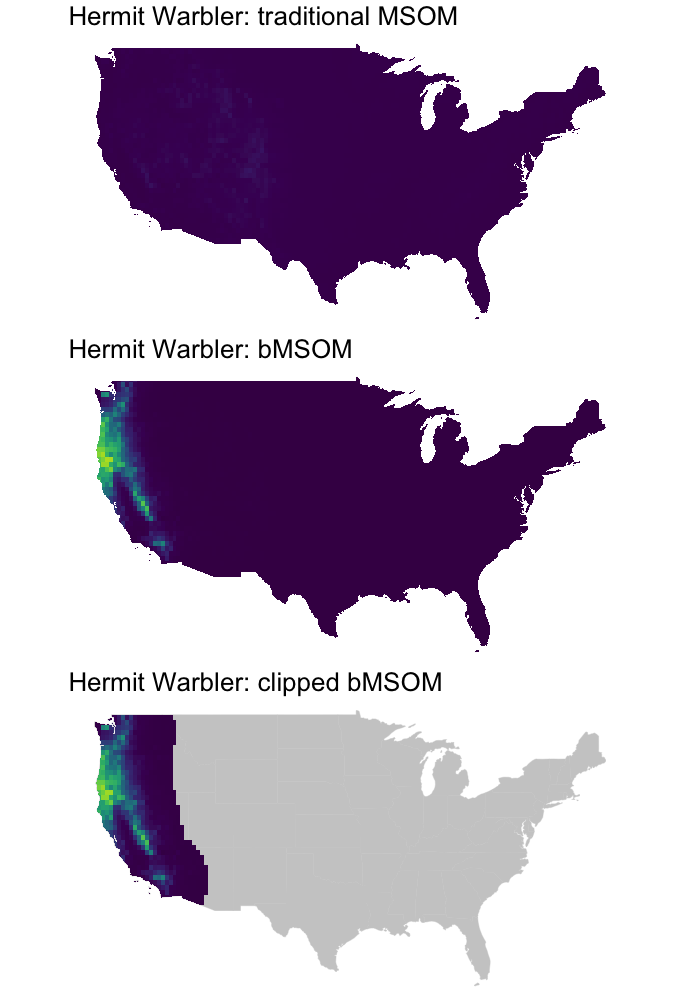

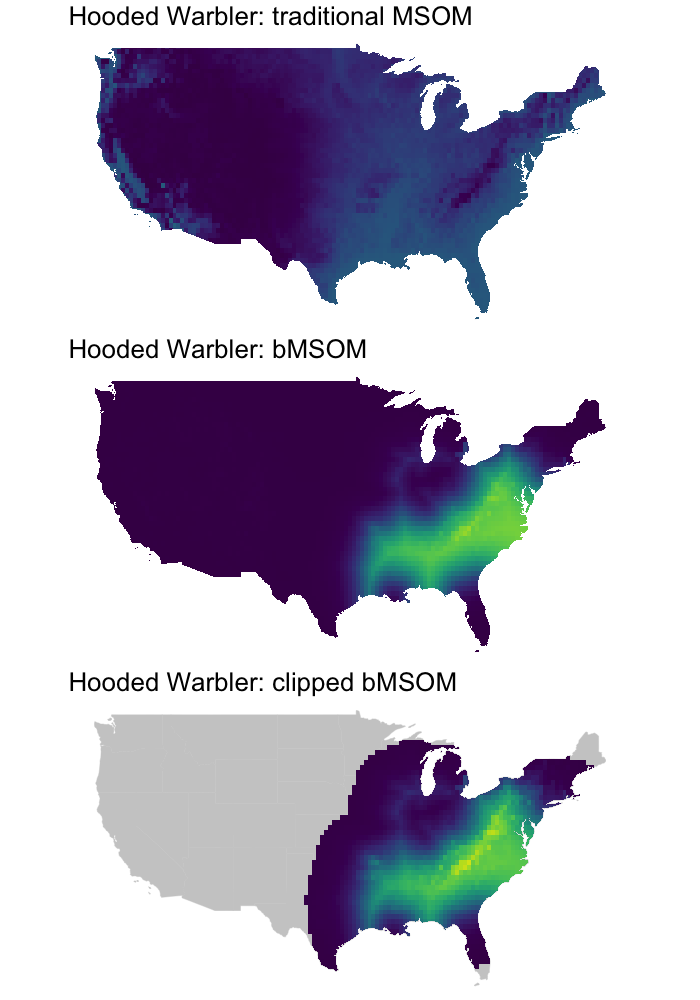


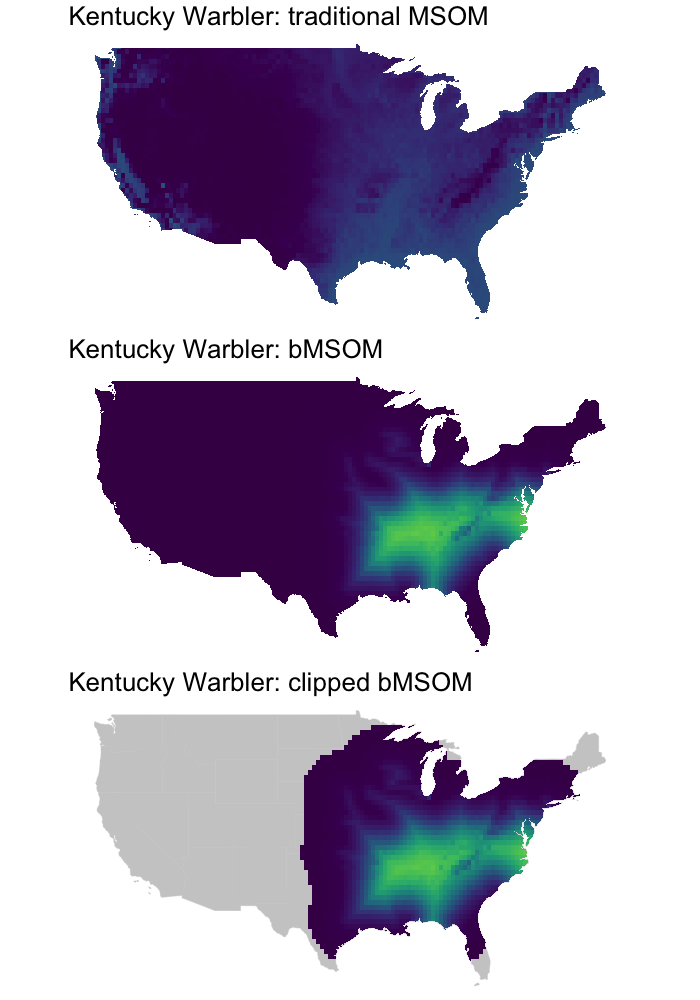

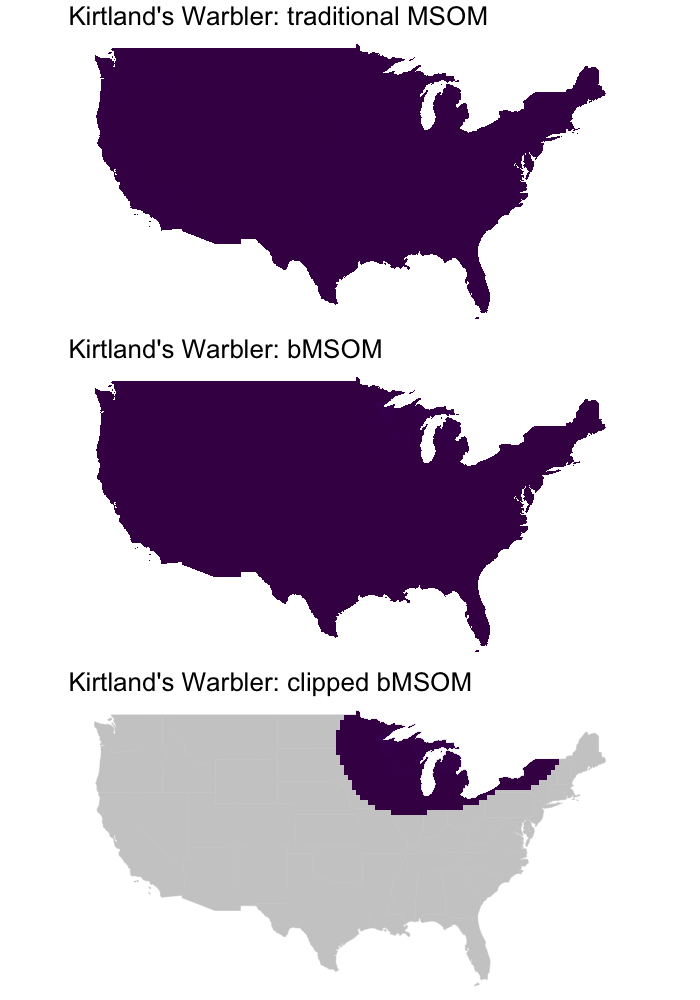


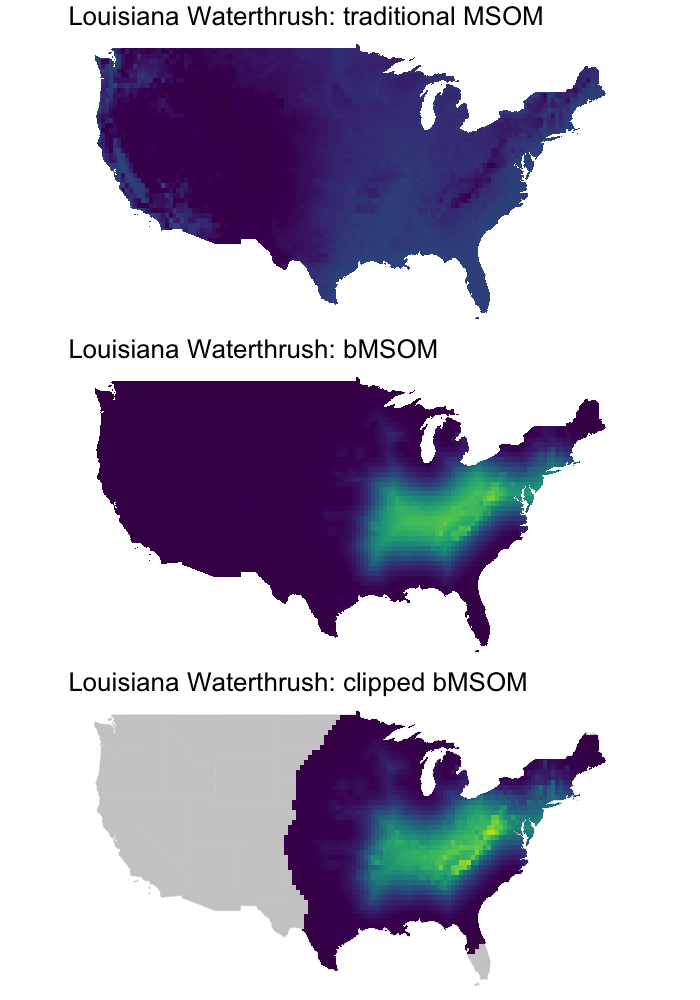

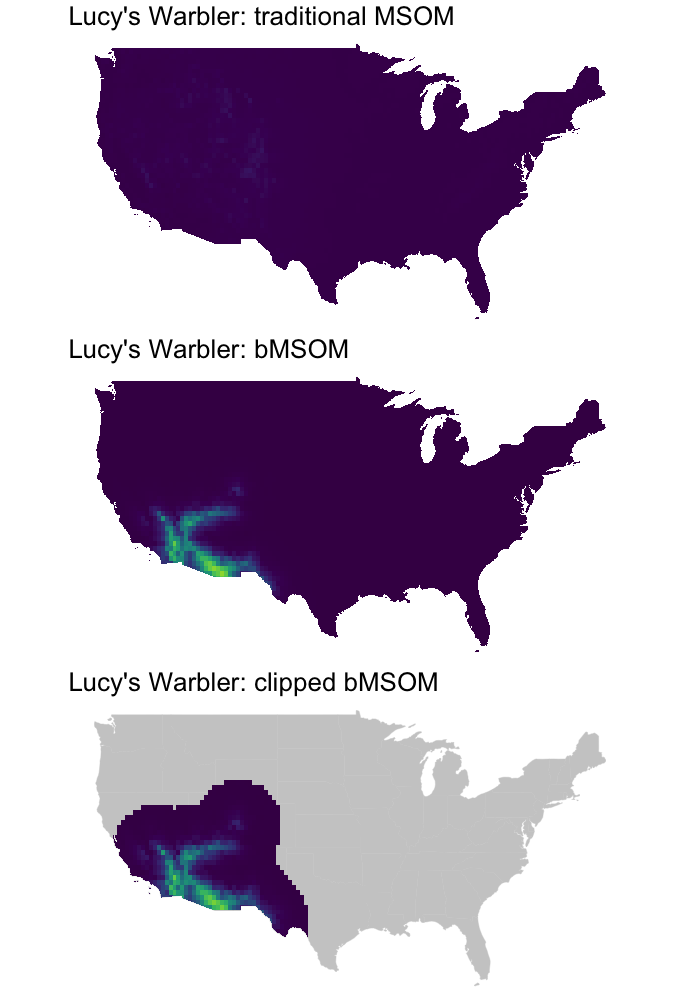


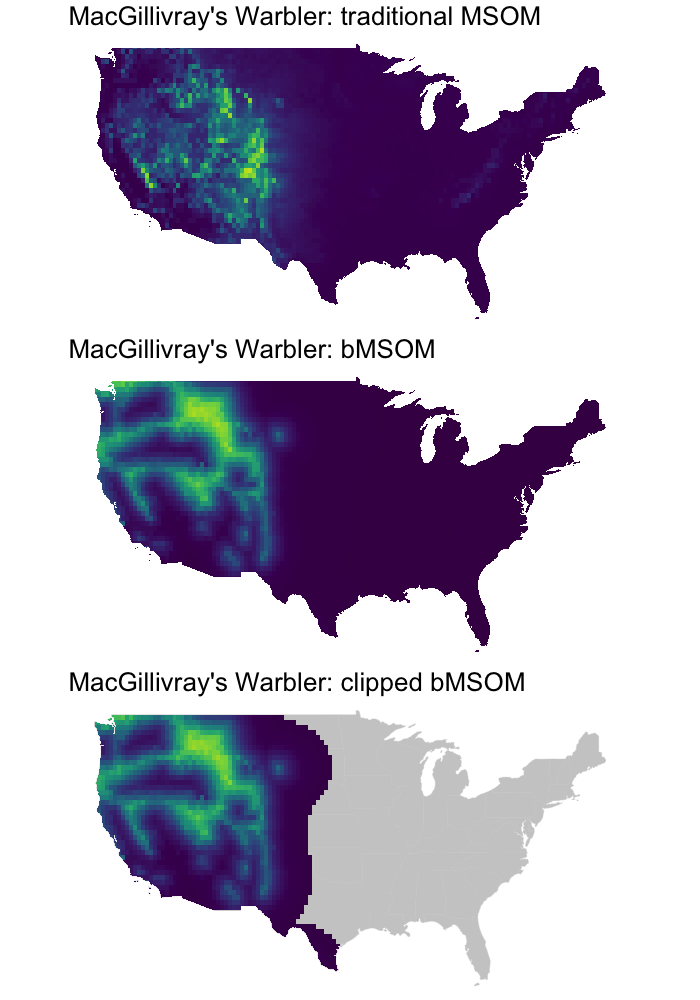

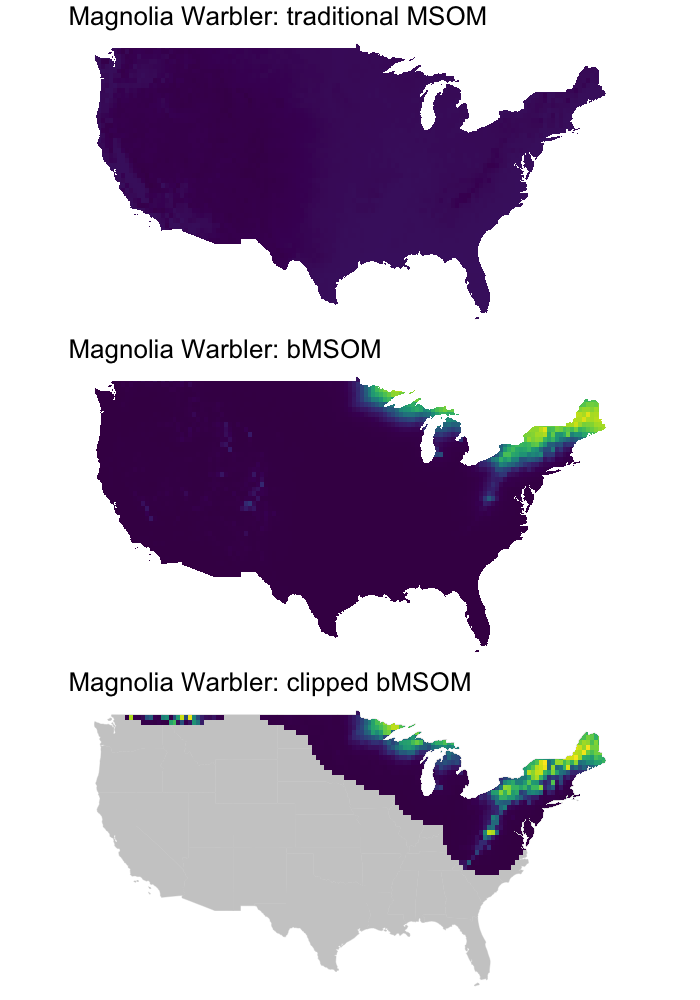


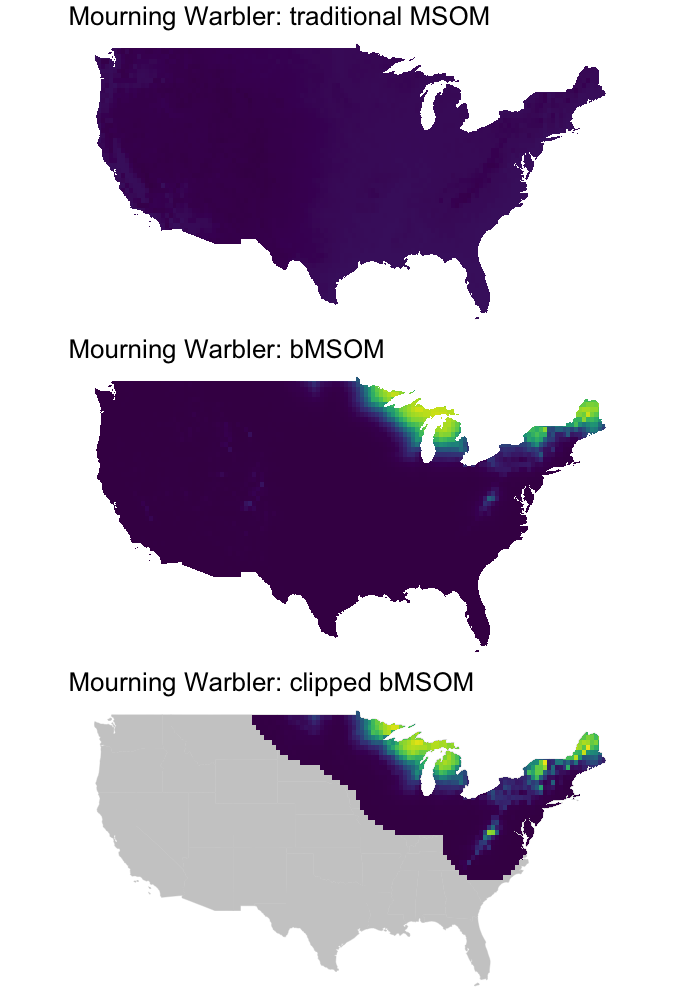

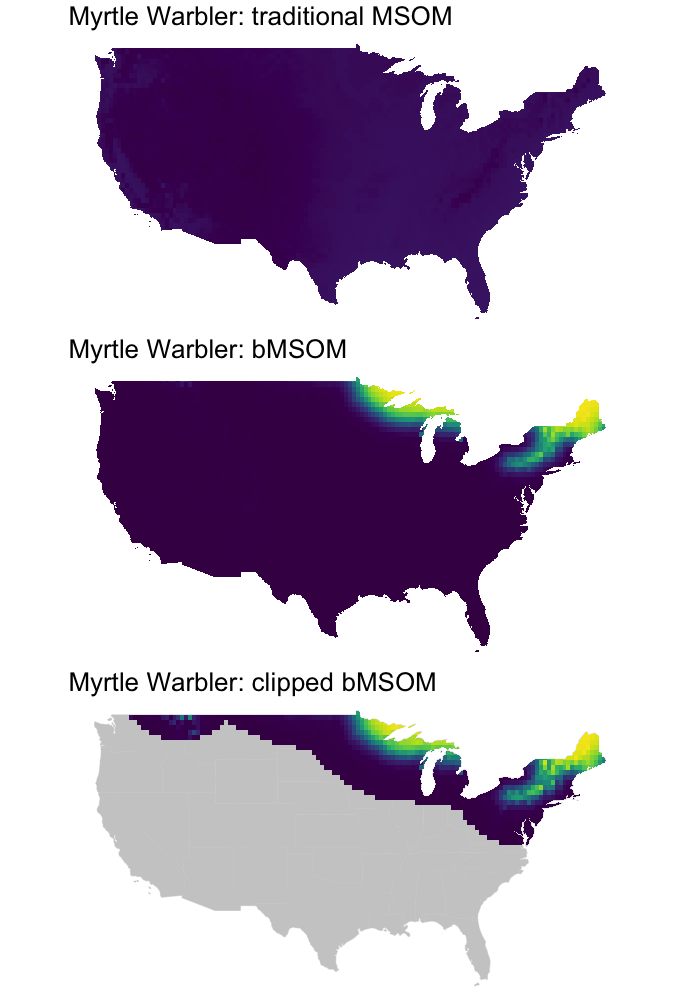


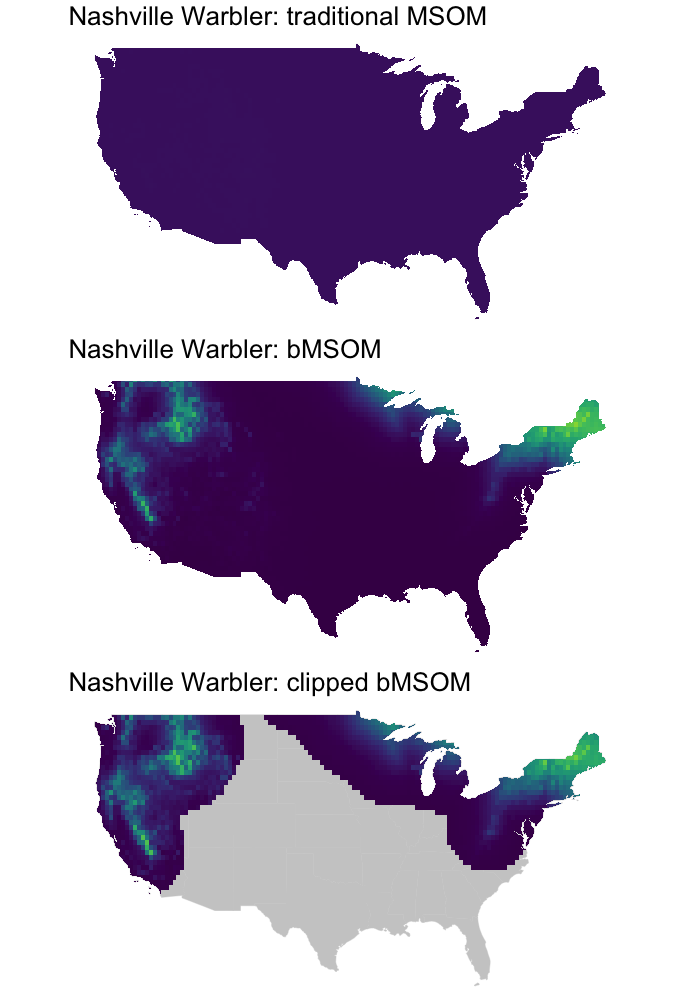

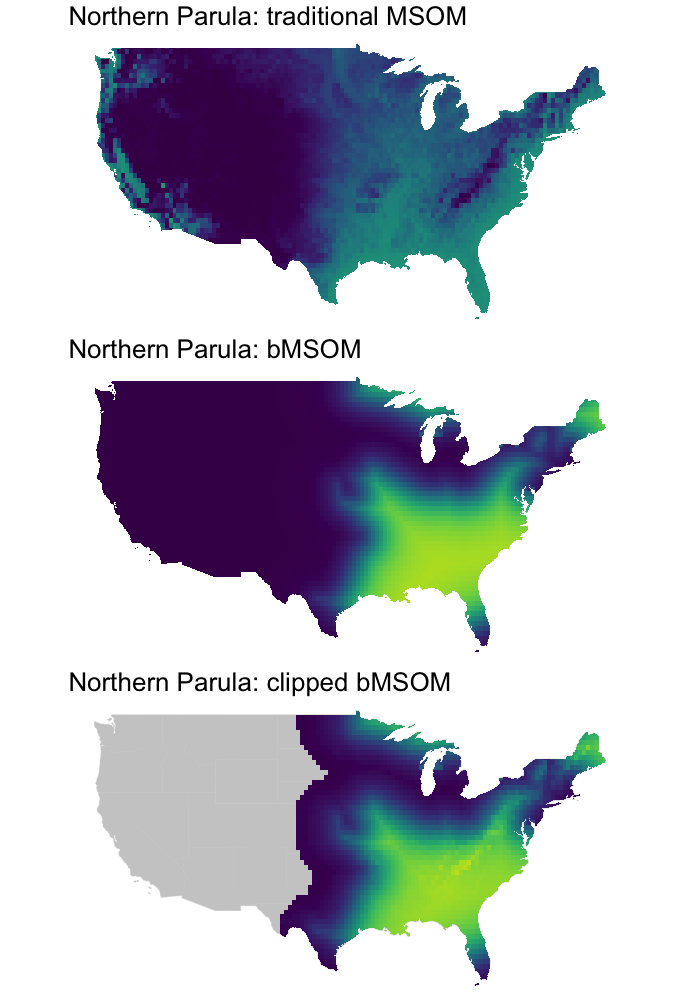


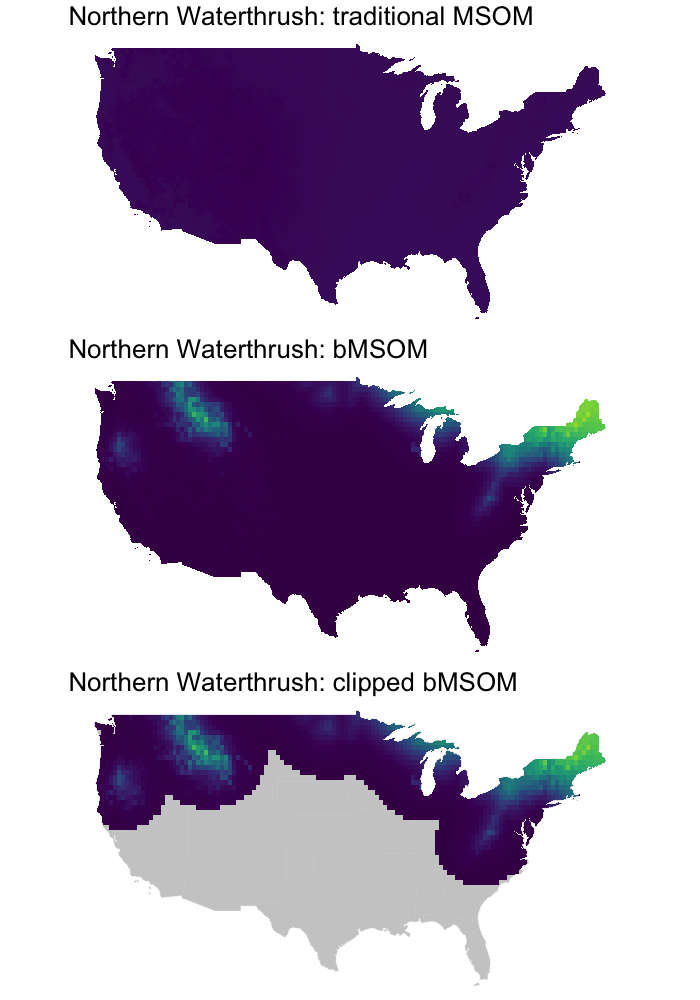

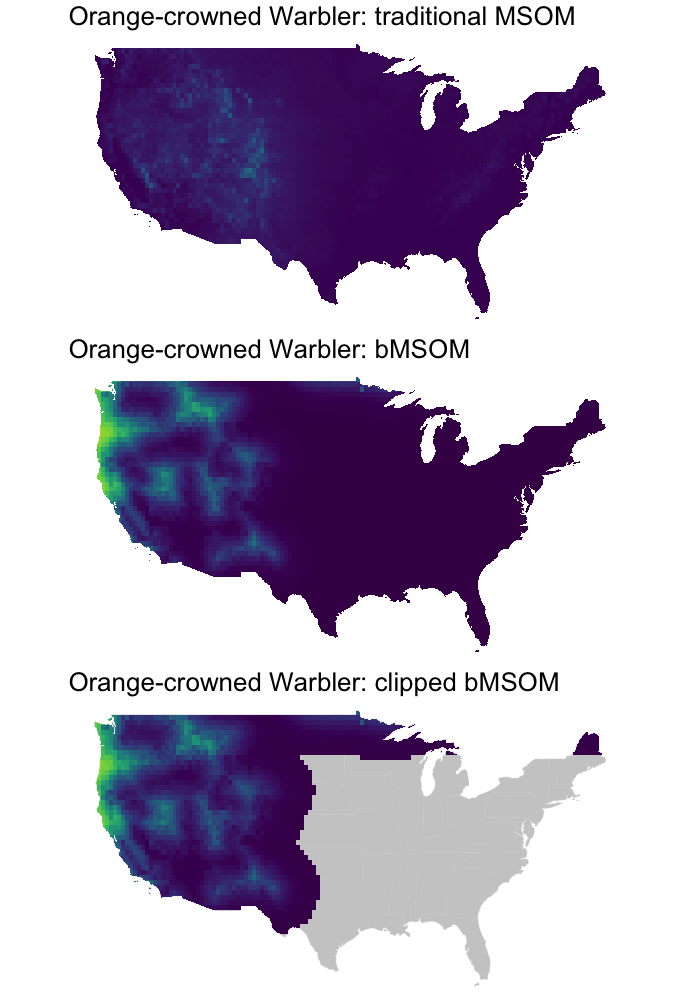


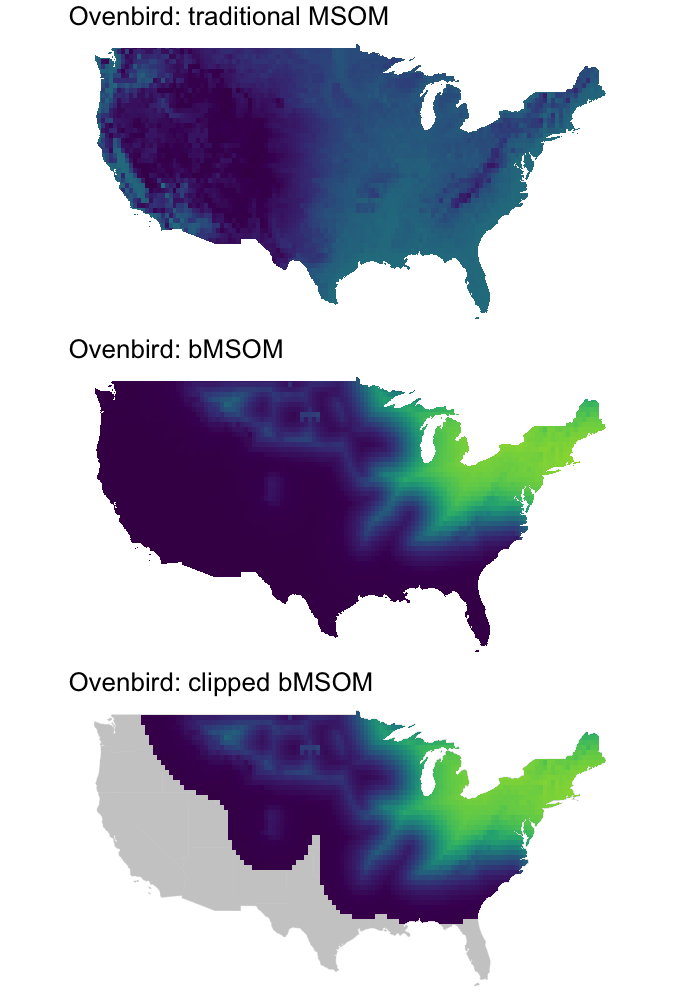

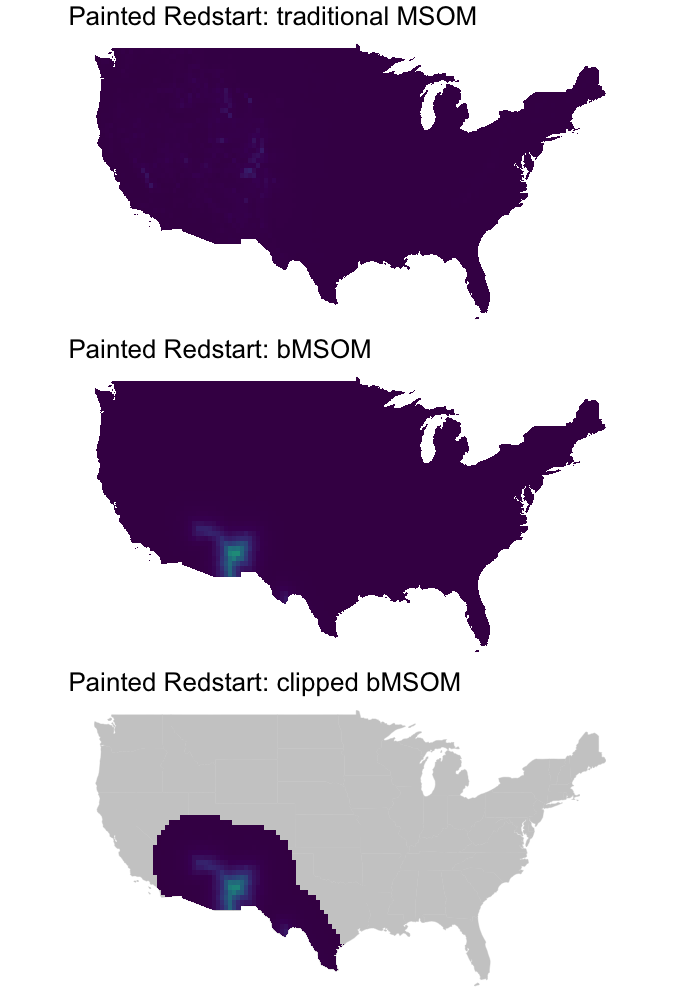


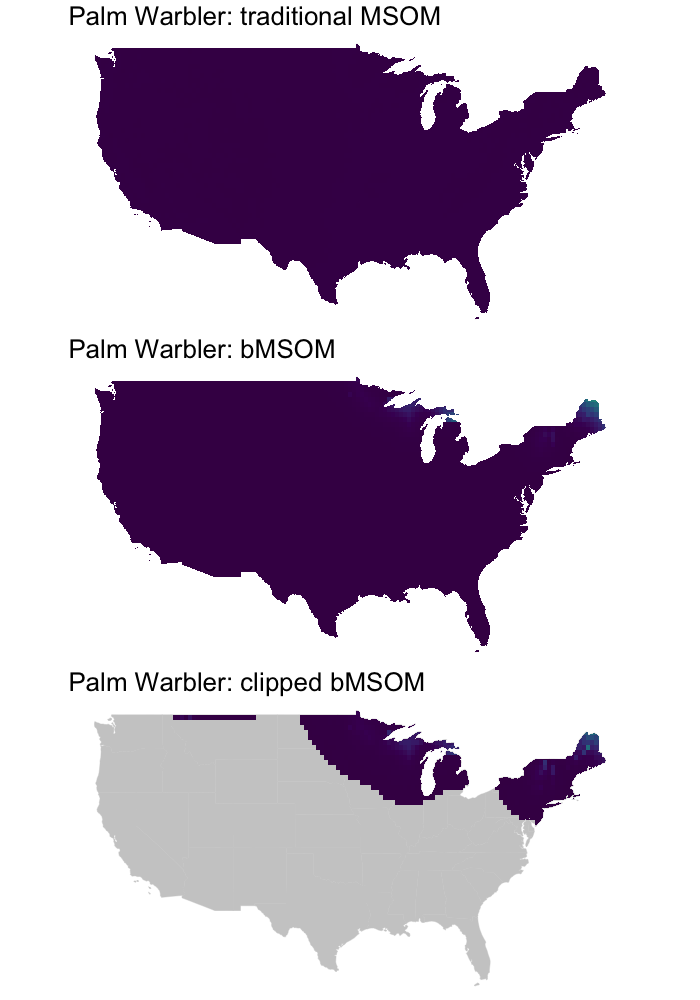

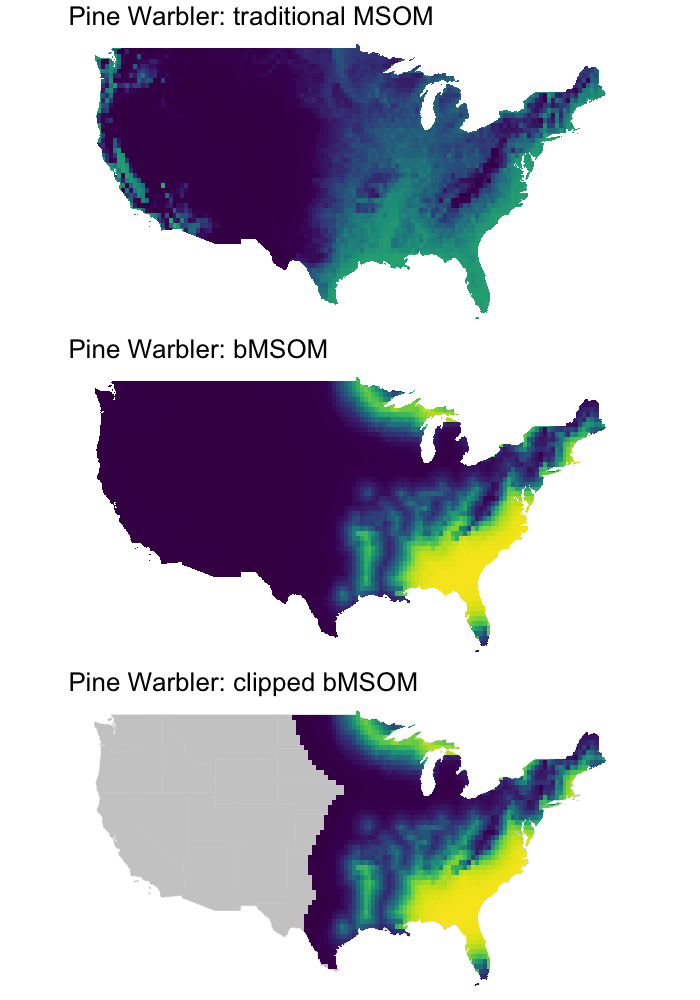


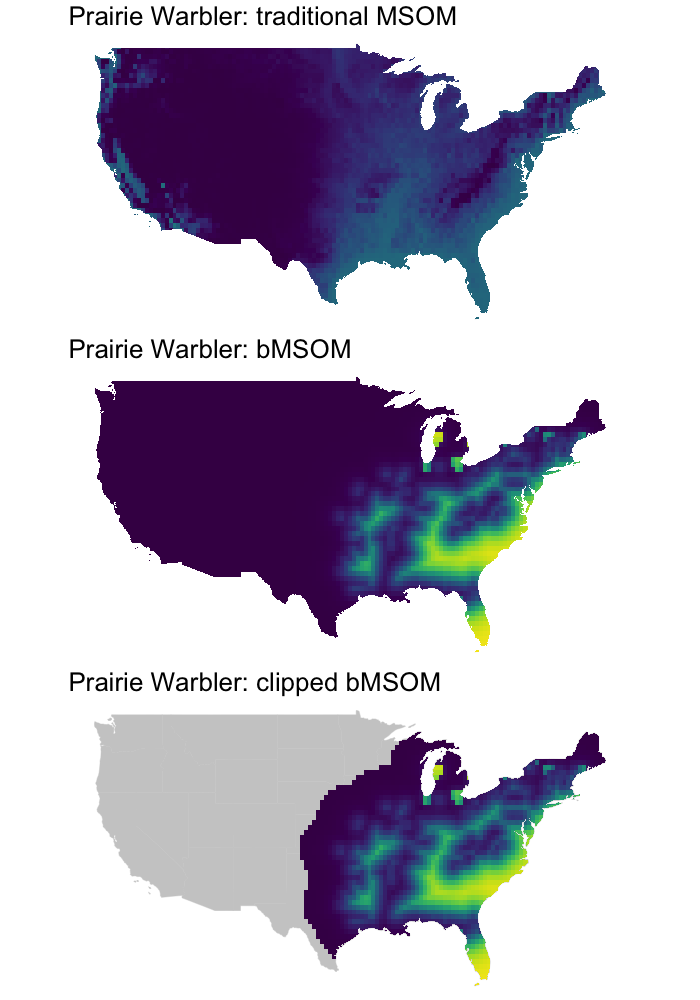

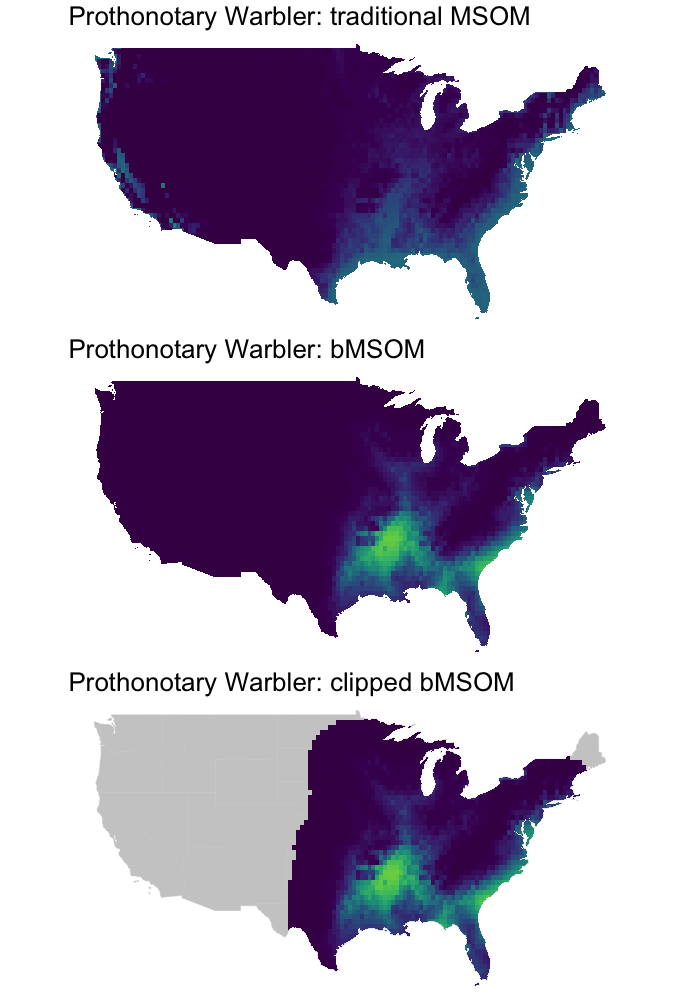


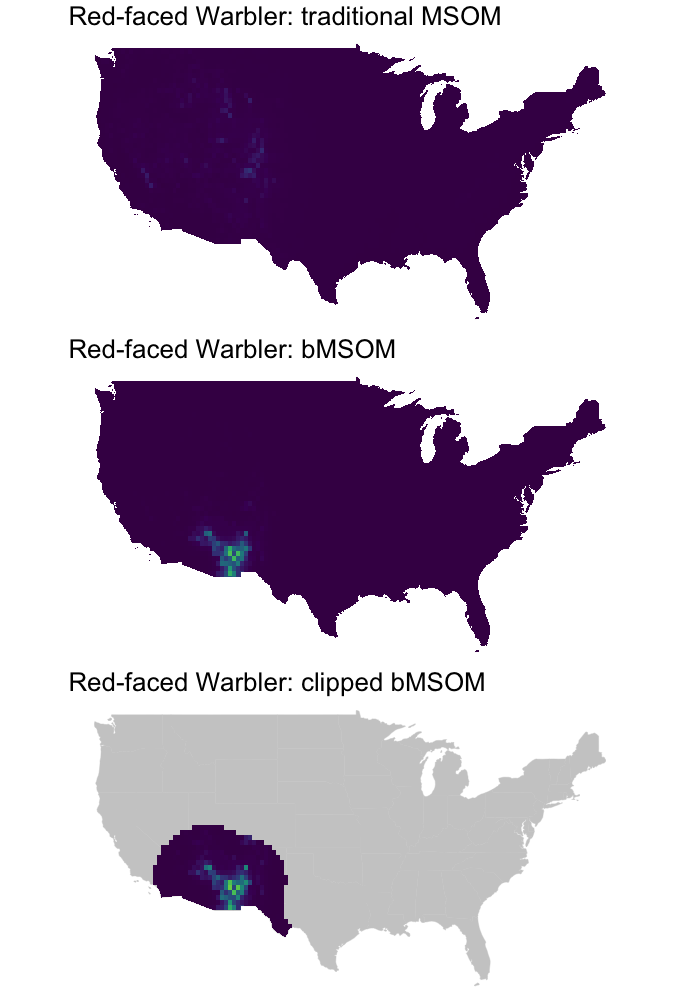

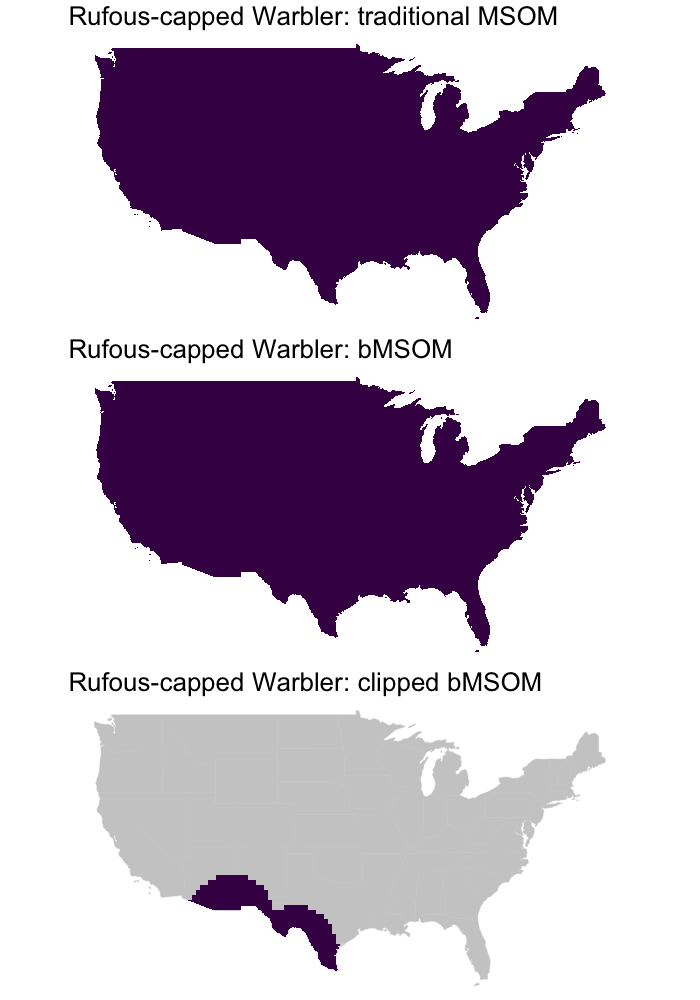


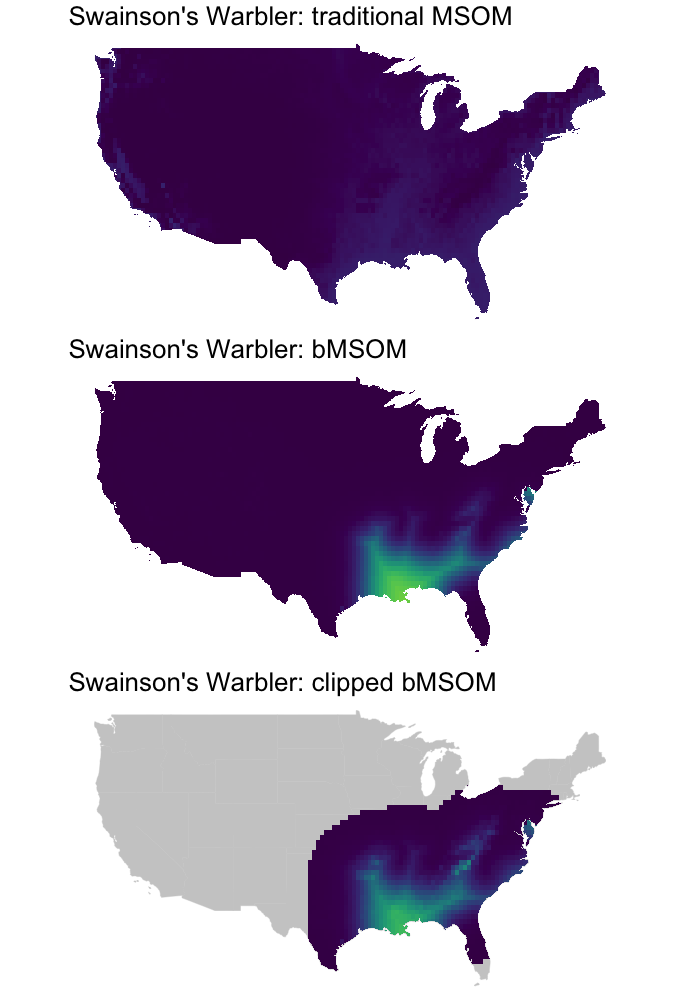

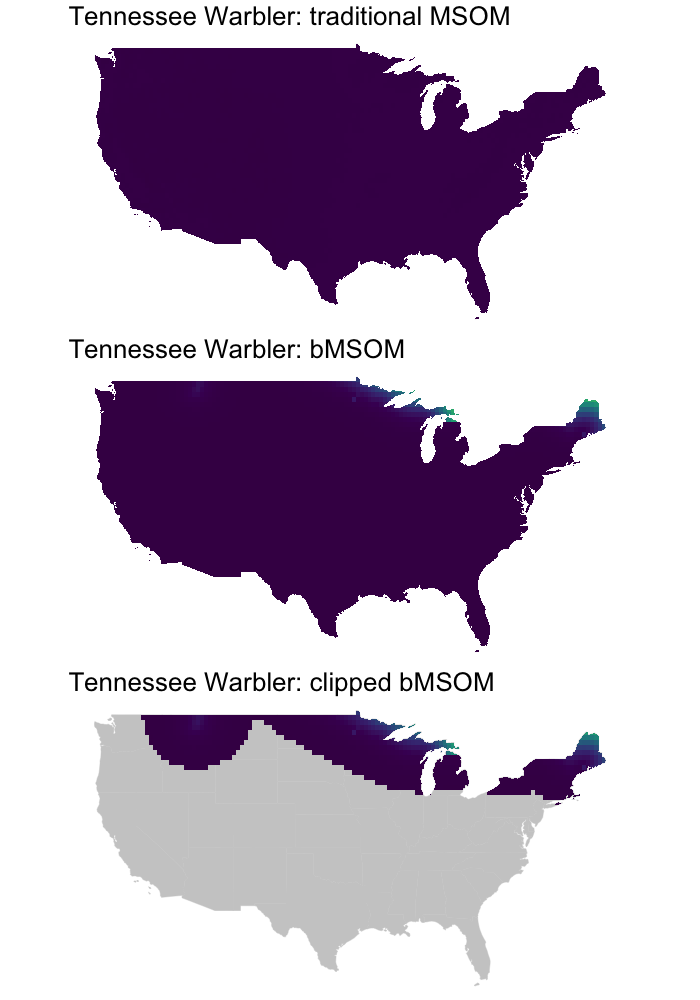


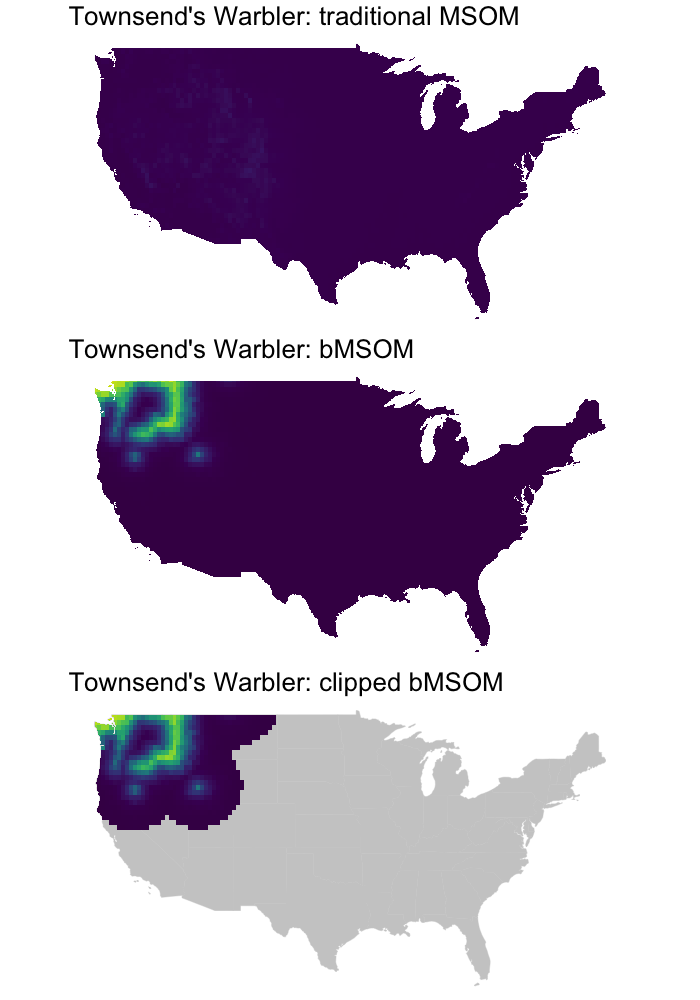

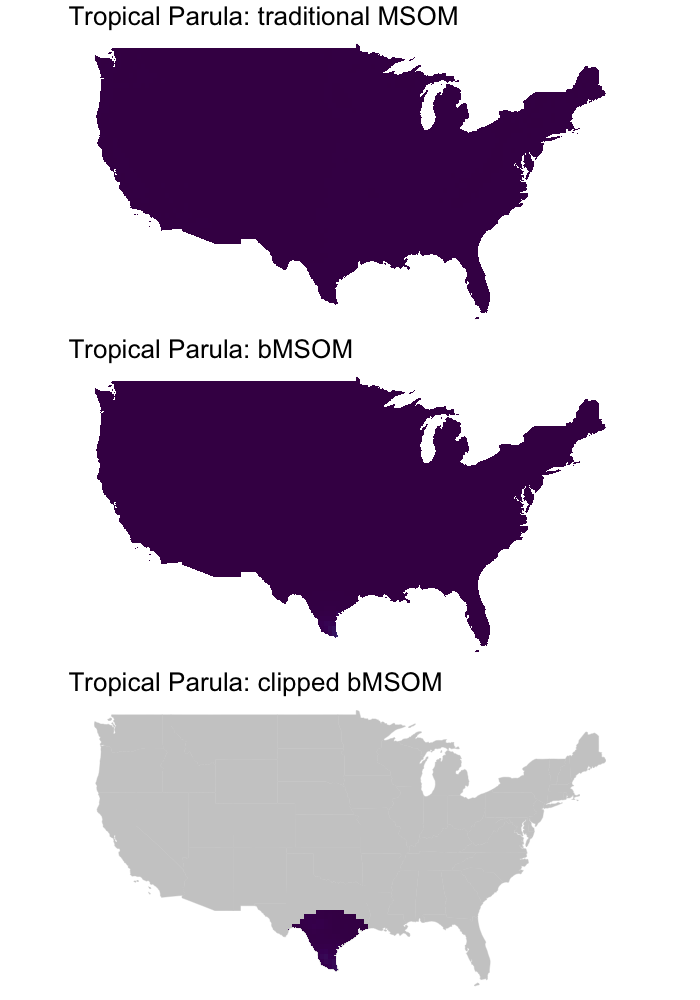


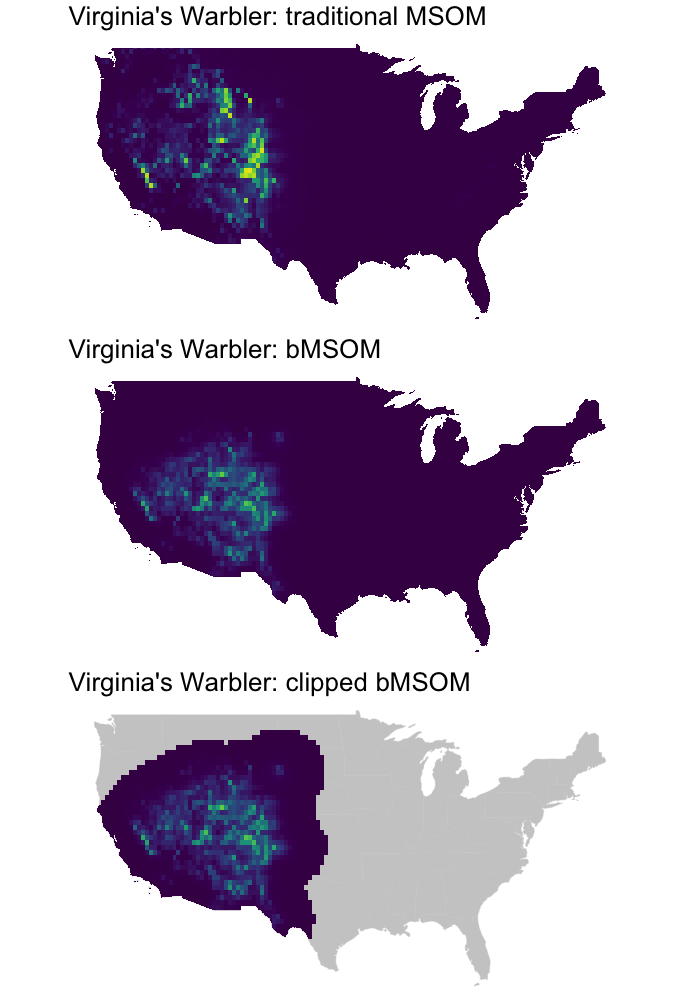

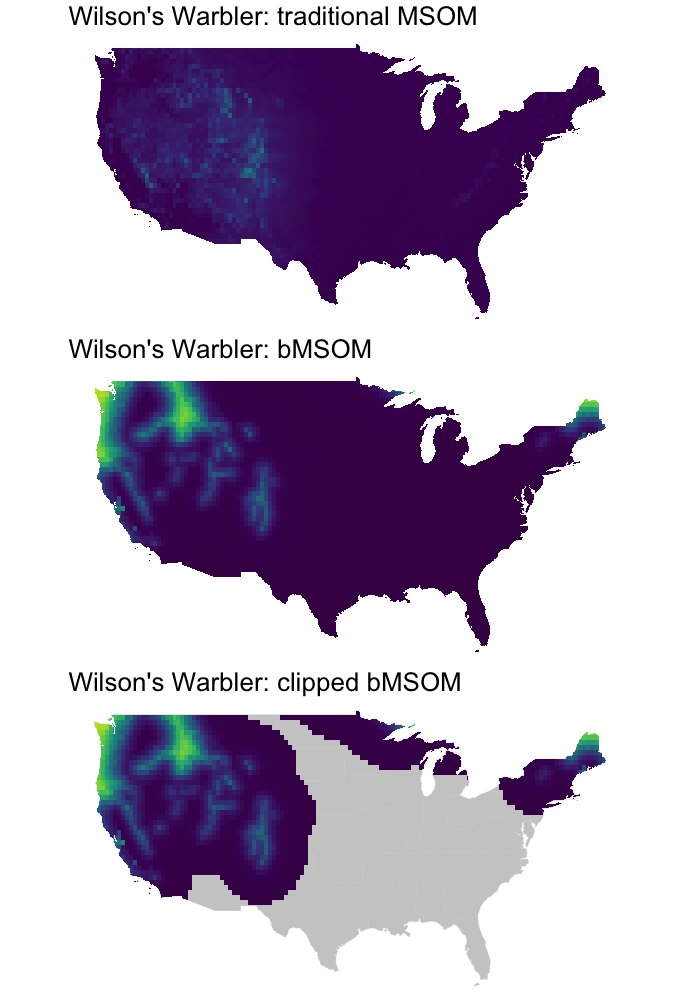


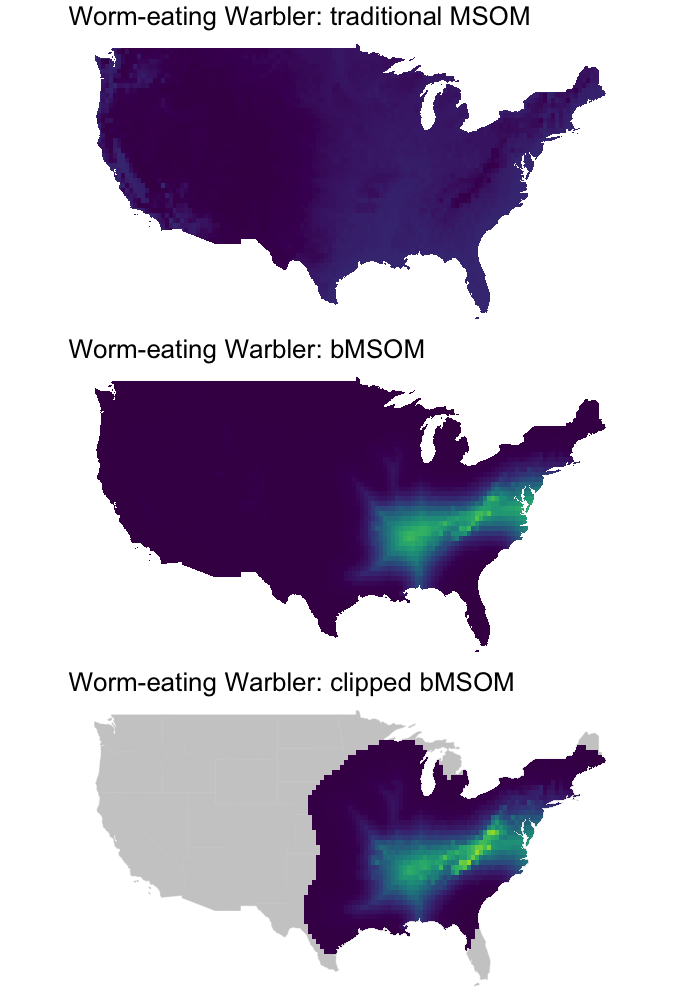

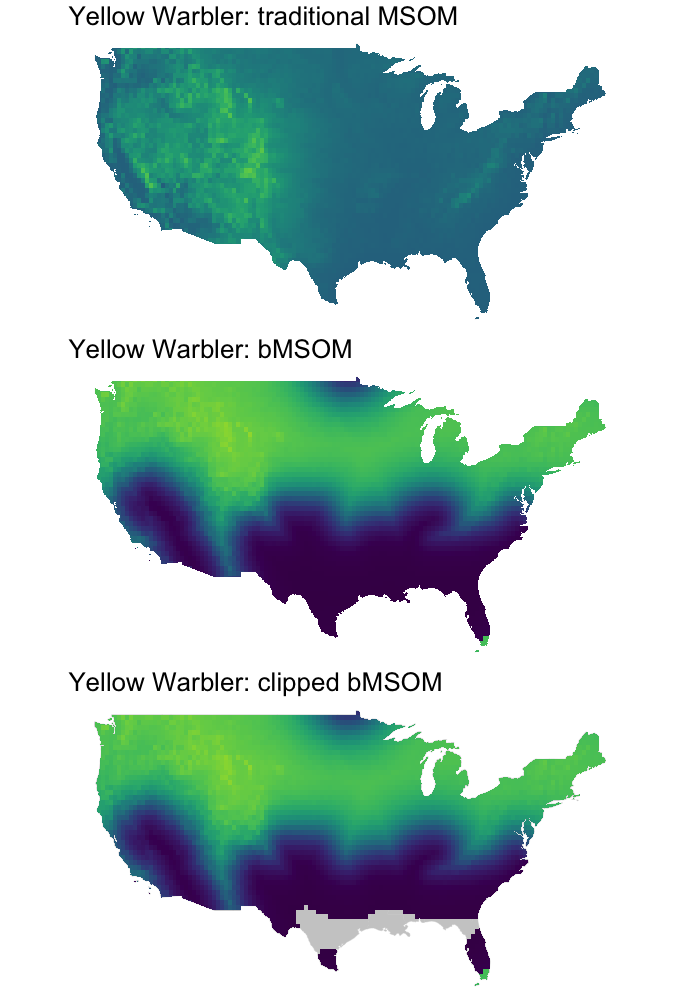


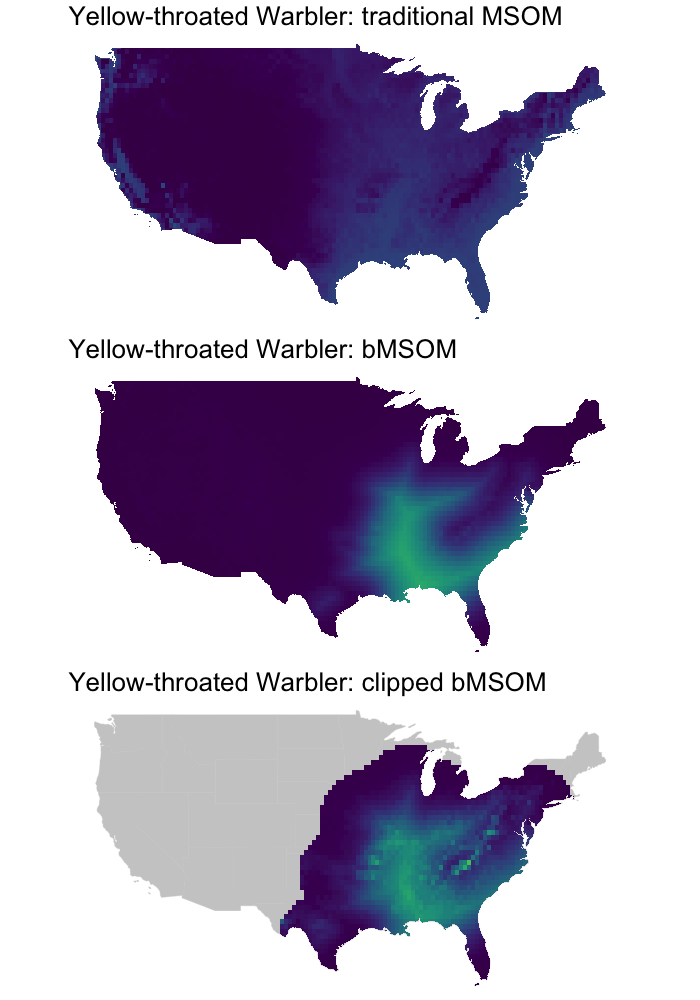

Supplement: Supplementary file 1 — Appendix S1 Supporting Information [file ECE3-12-e9328-s001.docx]
